# Epidemiological trends of urolithiasis in working-age populations: Findings from the global burden of disease study 1990–2021

**DOI:** 10.1371/journal.pone.0327343

**Published:** 2025-07-01

**Authors:** Weitao Yao, Xin Wei, Qiang Jing, Xiaobin Yuan, Fan liu, Xuhui Zhang

**Affiliations:** 1 Department of Urology, The First Clinical Medical School of Shanxi Medical University, Taiyuan, Shanxi, China; 2 Department of Urology, The First Clinical Medical School of Shanxi Medical University, Taiyuan, Shanxi, China; National Center for Chronic and Noncommunicable Disease Control and Prevention, Chinese Center for Disease Control and Prevention, CHINA

## Abstract

**Background:**

One of the most prevalent urinary disorders worldwide, urolithiasis has symptoms and a high rate of recurrence. It has placed a significant strain on the global economy and health care system, particularly among those aged 20–54. Comprehensive research on the global burden and evolving trends of urinary calculi in the 20–54 age range is lacking.

**Methods:**

Three important urolithiasis indicators—prevalence, incidence, and Disability-Adjusted Life Years (DALYs)—were used from the Global Burden of Disease (GBD) database between 1990 and 2021 for this study.Point estimates were provided, as well as 95% uncertainty intervals (UIs).. To assess trends in the burden of urolithiasis among the 20–54 age range, we used percentage change and estimated annual percentage change (EAPC). The time trends from 1990 to 2021 were thoroughly examined using joinpoint regression analysis. This method makes it possible to compute the annual percent change (APC) and average annual percent change (AAPC), as well as the 95% Confidence Intervals (CIs)that go with them.

**Results:**

The number of patients, cases, and DALYs linked to urinary stones among people aged 20–54 has significantly increased worldwide. For example, the number of urolithiasis cases increased by 48%, from 1,576,704 in 1990–2,335,010 in 2021. Comparably, the number of new cases increased by 47%, from 42,272,855 in 1990–62,269,033 in 2021. Between 1990 and 2021, the number of DALYs rose from 218,150–290,210, representing a roughly 33% increase. As of 2021, intermediate sociodemographic index (SDI) regions had the highest DALY rates, while medium-high SDI regions had the highest prevalence and incidence rates among the five SDI regions examined for this time period. Individuals between the ages of 50–54 had the greatest incidence rate within the designated cohort for that year, followed by those between the ages of 45–49 and 40–44. Across all age groups, there were noticeable gender differences, with males showing significantly greater rates than girls.

**Conclusions:**

Overall, over the past 32 years, the prevalence of urolithiasis among people aged 20–54 has increased dramatically worldwide, especially in low-SDI nations and among those aged 50–54. With the goal of reducing the social and medical cost and enhancing the working-age population’s quality of life and productivity, the study’s findings highlight the urgent need for focused intervention techniques to prevent and treat urolithiasis in this age group. For example, in middle – high SDI areas, encourage 3 liters of water and a low – salt diet daily, especially for the over - 50s. In middle SDI areas, enhance primary care diagnostics, like using portable ultrasound. Regular screenings should be set for males in high – risk jobs, such as heat – exposed ones. However, this GBD – based study has limitations like data uncertainty, insufficient local strategies, and lack of trend tracking.

## Introduction

Urolithiasis is among the most prevalent urinary disorders globally, with prevalence estimates varying from 1% to 13% across different regions of the world [1]. The incidence ranges from 7 to 13% in North America, 5–9% in Europe, and 1–5% in Asia [2–4]. With the passage of time, the number of patients with urinary stones is also increasing year by year. Recent evidence suggests that the changes in the prevalence of urolithiasis may be attributable to a combination of factors, including geographical location, social conditions, dietary habits, climatic conditions, comorbidities, genetic variations, and the adverse side effects of certain medications [5–7].Urolithiasis presents symptoms and a high recurrence rate that considerably diminish patients’ quality of life, while also heightening their risk for comorbidities such as fractures, renal dysfunction, obesity, diabetes, and cardiovascular diseases [1]. It also places a heavy burden on the world’s health care systems and the world economy [8,9]. One study showed that the estimated cost of treating urolithiasis in the United States was $3.79 billion in 2007 and is expected to increase by $1.24 billion per year by 2030 [10]. Urinary calculi have become a huge burden on public health.The incidence of stones varies by age, with lower incidence in children and the elderly [11,12]. Several studies have found that urolithiasis is more common in the working age population, with a second peak of urolithiasis occurring between 40 and 49 years of age for each gender since 2000. This research reveals that calcium – containing stone formation is more prevalent among individuals aged 20–29, with male patients reaching a plateau between 30–69 years old at 19.6%. In older age groups, men are three times more likely to develop calcium – containing stones than women. Since 2000, middle – aged adults (40–49 years old) of both genders have experienced a significant increase in calcium – containing stones. Calcium stone prevalence is notably higher in individuals over 40 compared to their proportion in the general population. Uric acid stones typically become more common starting in middle age (30–39 years old), while cystine stones are more prevalent at younger ages. The highest incidence rates were observed in women aged 20–29 and men aged 30–39 [13]. It is evident that the incidence of urinary calculi is highest among those aged 20–54, who are also the primary producers of social and economic goods. Urinary calculi’s acute pain, surgical necessity, and recurrence issues (which can occur up to 50% of the time) have a direct impact on productivity and labor force participation. The 20–54 age group is more receptive to health education than the elderly, and lifestyle changes like drinking water and nutrition (such as reducing salt and purine intake) can successfully lower the incidence rate. After the age of 60, the chance of developing chronic kidney disease increases fourfold if stones in patients aged 20–54 are not promptly removed, adding to the medical burden of an aging society.Therefore, it is necessary to comprehensively describe and analyze the overall incidence and changing trend of urinary stones in working age population. It is the strategic requirement for preserving the health of the workforce, maximizing the distribution of health resources, and achieving healthy aging, in addition to being the primary entry point for the prevention and control of disease burden.The Global Burden of Disease 2021 study is a systematic review of published and publicly available evidence on the incidence, prevalence and mortality of 369 diseases and injuries in 204 countries and territories and 21 regions for the period 1990–2021 [14]. Based on the latest data from the GBD2021 study, we analyzed the urinary stone incidence, prevalence and DALYs (disability-adjusted life years: It refers to all the years of healthy life lost from disease onset to death, including years of life lost due to premature death (YLL) and years lived with disability (YLD), the sum of YLL and YLD). for people aged 20–54 years at global, regional and country levels from 1990 to 2021, and compared the distribution and changes of urinary stone burden in different gender groups.

## Methods

### Data acquisition and download

A thorough assessment of the health hazards related to 369 illnesses and injuries, as well as 88 risk factors, in 204 nations and territories is provided by the 2021 Global Burden of Disease (GBD) research [15]. The most recent epidemiological data and improved standardized procedures are used in this study. The study used the GBD 2021 data to calculate prevalence, incidence, and disability-adjusted life years (DALYs) associated with urinary stones, as well as their 95% UI. All this datais accessible for free access through the Global Health Data Exchange (https://ghdx.healthdata.org/gbd-2021/sources)

### Socio‑demographic index (SDI)

The Institute for Health Metrics and Evaluation (IHME) introduced the Socio-demographic Index (SDI) in 2015 as a comprehensive tool to assess a country’s or region’s level of development, emphasizing the relationship between social development and population health outcomes. It combines the geometric mean of lagged per capita income, scaled from 0 to 1, the average educational attainment of those aged 15 and over, and the overall fertility rate of people under 25. A scale from 0 to 100 was created for the GBD 2021 by multiplying the SDI values by 100. A score of 0 denotes low income and degree of education with high fertility, while a score of 100 denotes high income and degree of education with low fertility. Five SDI groups—low, medium-low, medium, medium-high, and high—were assigned to the 204 nations and territories in 2021 [15].

### Disability‑adjusted life years

Disability-adjusted life years (DALYs), which represent the years of healthy life lost as a result of both premature mortality and disability, are a common metric used to evaluate health burden. DALYs = YLLs + YLDs is the formula that can be used to compute this. DALYs were equal to years lived with disability (YLDs) in this study because years of life lost (YLLs) were set to 0 because urinary stone-related fatalities could not be directly attributed in the GBD computation. To produce an estimate of the draw level, the computations were performed 500 times. Since simulation testing showed that this change had no discernible impact on the final estimates or their uncertainties, the number of computations was lowered from 1000 in earlier GBD iterations to 500 for GBD 2021. The 2.5th and 97.5th percentiles represent the 95% uncertainty interval, while the final estimate is based on the average of the 500 draws. Every stage of the estimating process takes uncertainty into consideration [15].

### Estimated annual percentage change and percentage change

In epidemiology, public health, and statistics, the term “estimated annual percentage change” (EAPC) is frequently used to describe the average yearly percentage change of an index over time, such as disease incidence, mortality, etc. It is frequently used to examine the rising or downward patterns of long-term trends and uses statistical models to predict the degree and direction of trend changes. EAPC > 0 indicates an annual average increase in the indicator, whereas EAPC < 0 indicates an annual average decrease.A reliable and popular metric for tracking changes in indicators like prevalence and incidence rates over predetermined time periods is the estimated annual percent change (EAPC) [16]. Estimating the dynamic trends in urinary stone incidence, prevalence, and DALYs between 1990 and 2021 was the goal of this study. By fitting the natural log of each observation to a straight line with time as a variable and calculating the slope of this line, the EAPC was determined using the natural log rate of the fitted regression model [17].


$$\begin{array}{c} y=\alpha +\beta x+\varepsilon \end{array}$$

$$\begin{array}{c} EAPC\;=100\times \left(exp\left(\beta \right)-1\right)\; \end{array}$$

The year is represented by X in the model, the natural logarithm of rates (such prevalence and incidence) by y, the intercept by α, the slope by β, and random error by ε. This fitted model was used to calculate the estimated annual percentage change’s (EAPC) 95% CI. The 95% confidence intervals were used to interpret the trend results: if the lower limit of the 95% CI was more than 0, a trend was considered upward; if the upper limit was less than 0, a trend was considered downward. It would indicate that the trend change was not statistically significant if the 95% CI contained 0 [18]. Additionally, this study used percentage changes to show how prevalence, incidence, and DALYs changed between 1990 and 2021.


$$\begin{array}{c} Percentage\;change=(2021\;cases-1990\;cases)/1990\;cases \end{array}$$

Data cleaning, calculations, and plotting were performed with the use of R software, version 4.4.1. Visualizations were created using the ggplot2 package.

### Joinpoint regression analysis

In order to evaluate temporal trends in disease frequency or death, we employed a connective-point regression model, a statistical technique frequently used in epidemiologic investigations [19]. One statistical technique for identifying turning points (“joining points”) of trends in time-series data is joinpoint regression analysis. By splitting time series into several linear intervals and determining the points at which trends change considerably, it is frequently used in epidemiology, public health, and economics to examine long-term trend changes in morbidity, mortality, or economic indicators.Important shift points in time-series data on the prevalence of urinary stones from ages 20–54 were statistically defined and expertly detected by the model using global, continental, and national scale data. To illustrate changes in prevalence throughout the period shown, the model makes it easier to compute the annual percent change (APC) and the 95% confidence interval (CI) that goes with it. Additionally, the average annual percent change (AAPC), which takes into account aggregated trend data for the study period from 1990 to 2021, was computed in order to thoroughly evaluate the patterns that were detected. An upward trajectory across the designated interval is shown statistically by an APC or AAPC estimate where the lower bound of its 95% CI is greater than zero. On the other hand, a decreasing trend is indicated by an APC or AAPC estimate plus a 95% CI upper limit below zero. The trend is said to be stable when the 95% CI of APC or AAPC is zero.

### Statistics analysis

Prevalence, incidence, mortality, and disability-adjusted life years are expressed as predictions per 100 000 population, including their 95% UI. All analyses and graphical presentation procedures were performed with the use of statistical software R, version 4.4.1.

## Results

### Global level

**Fig 1:**
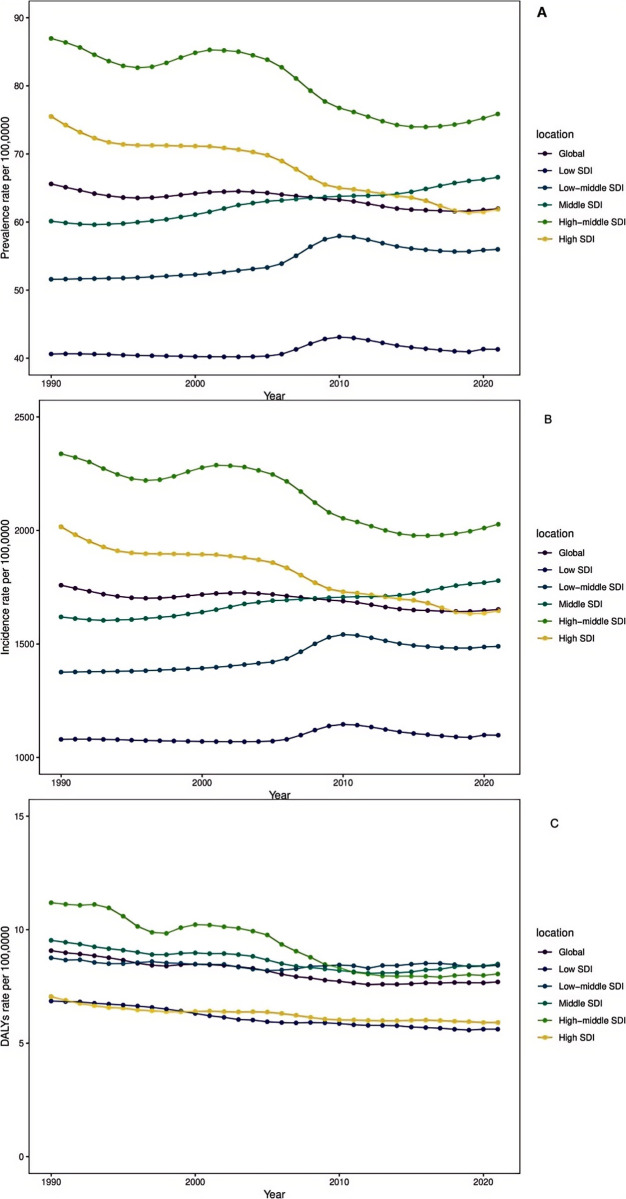
Temporal trend of migraine burden in WCBA in global and 5 territories. (A) ~(C) The rates of prevalence, incidence, and DALYs from 1990 to 2021. DALYs = Disability-Adjusted Life Years.

**Fig 2:**
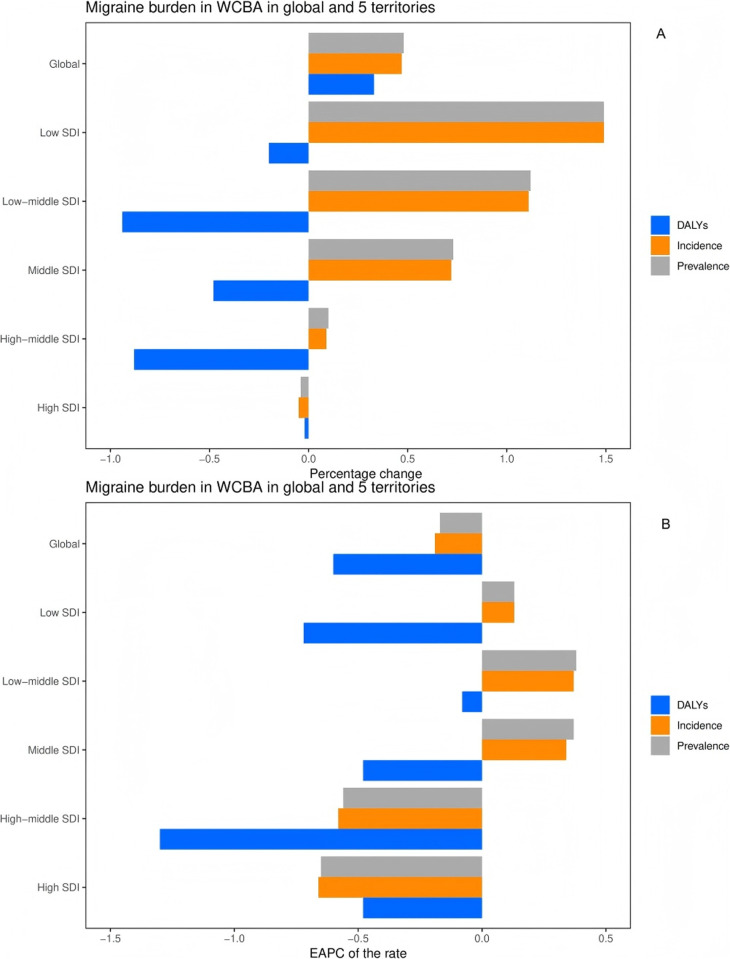
Temporal trend of migraine burden in 20-54years in global and 5 territories. (A) Percentage change in cases of prevalent, incident, and DALYs in 1990 and 2021. (B) The EAPC of prevalence, incidence, and DALY rates from 1990 to 2021. EAPC = Estimated Annual Percentage Change, DALYs = Disability-Adjusted Life Years.

Globally, the number of patients, the number of cases, and the number of DALYs associated with urinary stones in the 20−54 age group increased significantly.For example, urolithiasis cases increased from 1.58 million in 1990 to **2.34 million** in 2021, a percentage change of 48%; The number of new cases increased from **42.27 million** in 1990 to **62.27 million** in 2021, with a percentage change of 47%. DALYs cases increased from **0.22 million** in 1990 to **0.29 million** in 2021, a percentage change of 33% (Table 1 and S1 and S2). This indicates that the burden of urinary stones among persons 20–54 years of age continues to increase worldwide (Table 1 and S1–S3 and Figs 1−2 and S1–S3). However, from 1990 to 2021, the global prevalence, incidence and DALY rate of urinary stones showed a downward trend among the 20−54 age group, and the EAPC was −0.17 (95%CI:-0.2--0.14) and −0.19 (95%CI:-0.22--0.15), respectively. −0.6 (95%CI:-0.67--0.54) (Table 1 and S1 and S2, Fig 1A ~ C). While the age-standardized rate shows a downward trend (1990–2021), this suggests that the disease burden of urinary calculi in people aged 20–54 years worldwide is still increasing. This suggests that while aging and population growth may be the primary causes of the absolute increase, the age-standardized risk is actually declining. This could imply that aging and population growth worldwide, rather than elevated personal risk, are the causes of the growing burden of disease.

**Table 1:** The prevalence of urinary stones and rates among 20-54years in 1990 and 2021, and the trends from 1990 to 2021.

location	Prevalent cases			Prevalent rates		
	1990 (95% UI)	2021 (95% UI)	percentage change (100%)	1990_per 100 000(95% UI)	2021_per 100 000(95% UI)	EAPC(95% CI)
Andean Latin America	13641.64 (10340.71-17743.4)	32130.35 (25316.93-40396.63)	1.36	86.99 (65.94-113.15)	98.7 (77.77-124.09)	0.55 (0.47-0.63)
Australasia	6632.33 (4812.97-8830.34)	9712.37 (7094.43-12938.02)	0.46	65.9 (47.83-87.75)	66.59 (48.64-88.71)	0.01 (−0.05-0.06)
Caribbean	6980.96 (5227.01-9169.39)	12941.37 (9628.09-17067.11)	0.85	43.95 (32.91-57.73)	56.42 (41.97-74.41)	0.88 (0.82-0.94)
Central Asia	23099.71 (17806.24-29895.51)	38566.3 (29738.82-50010.27)	0.67	77.69 (59.89-100.54)	82.7 (63.77-107.24)	0.28 (0.22-0.33)
Central Europe	37361.34 (28545.98-49353.64)	28177.67 (21630.76-36450.55)	−0.25	63 (48.14-83.22)	51.53 (39.56-66.66)	−0.65 (−1.06--0.23)
Central Latin America	30951.3 (22949.85-41023.79)	74020.83 (56836.28-95923.81)	1.39	45.38 (33.65-60.15)	59.24 (45.49-76.77)	1.06 (0.75-1.37)
Central Sub-Saharan Africa	5609.15 (4214.43-7390.99)	16435.49 (12342.21-21681.74)	1.93	27.75 (20.85-36.56)	30.24 (22.71-39.9)	0.28 (0.24-0.31)
East Asia	363629.48 (272911.59-477666.61)	398221.63 (300760.52-522276.37)	0.1	59.77 (44.86-78.52)	54.13 (40.88-70.99)	−0.54 (−0.72--0.37)
Eastern Europe	218017.52 (169446.46-280782.63)	181003.68 (137307.79-234287.09)	−0.17	197.62 (153.59-254.51)	183.72 (139.37-237.81)	−0.31 (−0.38--0.23)
Eastern Sub-Saharan Africa	24253.64 (18450.39-31857.76)	61368.88 (46995.15-80034.29)	1.53	35.79 (27.23-47.01)	35.79 (27.41-46.67)	−0.13 (−0.16--0.1)
Global	1576704.02 (1189918.14-2050374.76)	2335010.67 (1789652.77-3011581.44)	0.48	65.6 (49.51-85.3)	61.94 (47.48-79.89)	−0.17 (−0.2--0.14)
High-income Asia Pacific	69092.75 (48702.04-93392.12)	71267.73 (51908.56-95627.11)	0.03	78.44 (55.29-106.02)	84.69 (61.68-113.63)	0.24 (0.2-0.29)
High-income North America	120490.75 (87976.31-161646.63)	75780.83 (61277.52-95897.81)	−0.37	85 (62.06-114.04)	45.09 (36.46-57.05)	−2.36 (−2.66--2.07)
High-middle SDI	452912.52 (344305.36-593687.43)	496179.72 (376310.69-646085.54)	0.1	86.96 (66.11-113.99)	75.87 (57.54-98.79)	−0.56 (−0.65--0.46)
High SDI	333435.64 (243820.41-444243.36)	319561.91 (242667.53-420797.41)	−0.04	75.48 (55.19-100.56)	61.89 (47-81.5)	−0.65 (−0.71--0.6)
Low-middle SDI	242287.73 (183342.15-315918.1)	512644.57 (389957.46-665919.08)	1.12	51.6 (39.04-67.28)	55.99 (42.59-72.73)	0.38 (0.3-0.46)
Low SDI	74907.63 (56533.66-97749.23)	186352.38 (140638.96-243007.81)	1.49	40.62 (30.66-53)	41.31 (31.18-53.87)	0.13 (0.06-0.2)
Middle SDI	471893.93 (357924.19-613987.94)	818595.94 (628062.05-1056541.33)	0.73	60.13 (45.61-78.24)	66.58 (51.08-85.93)	0.37 (0.34-0.39)
North Africa and Middle East	54554.24 (40120.64-71925.99)	147156.65 (106628.63-195819.23)	1.7	40.66 (29.91-53.61)	47.43 (34.37-63.11)	0.5 (0.49-0.51)
Oceania	1102.49 (818.78-1474.27)	2820.19 (2086.91-3657.22)	1.56	40.8 (30.3-54.55)	44.72 (33.09-57.99)	0.31 (0.24-0.37)
South Asia	258493.82 (194493.53-336950.14)	593809.14 (448009.09-771962.57)	1.3	56.68 (42.65-73.89)	64.91 (48.97-84.39)	0.7 (0.54-0.85)
Southeast Asia	138294.46 (105152.16-176866.94)	266248.39 (203143.28-342870.21)	0.93	68.04 (51.73-87.01)	75.11 (57.31-96.73)	0.29 (0.26-0.32)
Southern Latin America	21694.05 (15620.03-28813.03)	38855.37 (30241.37-49518.84)	0.79	97.56 (70.24-129.57)	116.09 (90.36-147.96)	0.3 (0.19-0.41)
Southern Sub-Saharan Africa	7706.3 (5763.48-10127.17)	14404.59 (10683.98-18953.65)	0.87	35.79 (26.77-47.03)	36.65 (27.18-48.22)	0.04 (−0.02-0.09)
Tropical Latin America	19129.98 (14804.06-24669.7)	62801.62 (49747.73-80689.76)	2.28	28.07 (21.72-36.2)	53.84 (42.65-69.18)	1.72 (1.22-2.22)
Western Europe	134627.5 (99307.53-178619.17)	153819.66 (117555.7-202417.95)	0.14	71.25 (52.56-94.53)	78.26 (59.81-102.99)	0.6 (0.38-0.81)
Western Sub-Saharan Africa	21340.62 (15960.79-28171.57)	55467.94 (41430.14-72713.77)	1.6	29.96 (22.41-39.55)	29.33 (21.91-38.45)	−0.1 (−0.14--0.05)

### SDI regional level

In 2021, the prevalence, incidence and absolute number of DALYs of urinary stones in 20–54 years old group were the highest in middle SDI **0.82 million** (95%UI: 0.63 **million**-1.06 **million**), **21.87 million** (95%UI:16.64 **million**-28.10 **million**) and **0.10 million** (95%UI:0.08 **million**-0.14 **million**), respectively. In 2021, the highest prevalence, incidence, and DALYs rates were found in moderately high SDI regions, while the highest DALYs rates were found in moderate SDI regions (Table 1 and S1, S2). The prevalence rate and incidence rate increased with the increase of SDI, but reached the highest level in the middle and high SDI areas. DALYs increased gradually with the increase of SDI, and reached the highest level in the middle SDI area and then decreased, with the lowest SDI area having the largest percentage of change (about 150%). In contrast, regions with high SDI showed the smallest percentage change (about −4% to −5%) (Table 1 and S1, S2, Fig 2A and 2B). It is noteworthy that from 1990 to 2021, the prevalence and incidence of SDI in the central and low-middle regions showed a rapid increase, with an EAPC of 0.37 (95%CI:0.34–0.39), respectively. 0.38 (95% CI: 0.3 0.46) and 0.37 (95% CI: 0.34 0.39), 0.38 (95% CI: 0.3 0.46) (Table 1 and S1, S2, Fig 2A and 2B). Therefore, the prevalence and incidence of urinary calculi in the 20–54 age group were the highest in the middle and high SDI regions, but the rate of increase was the fastest in the middle SDI region. Incidence and prevalence rise with SDI, peaking in moderate to high SDI regions. DALY rates peak in moderate SDI areas, then decline. Low-SDI regions see the largest increase (+150%), while high-SDI areas show minimal change due to better prevention. Moderate-SDI regions carry the heaviest and fastest-growing disease burden.

### GBD regional level

**Fig 3:**
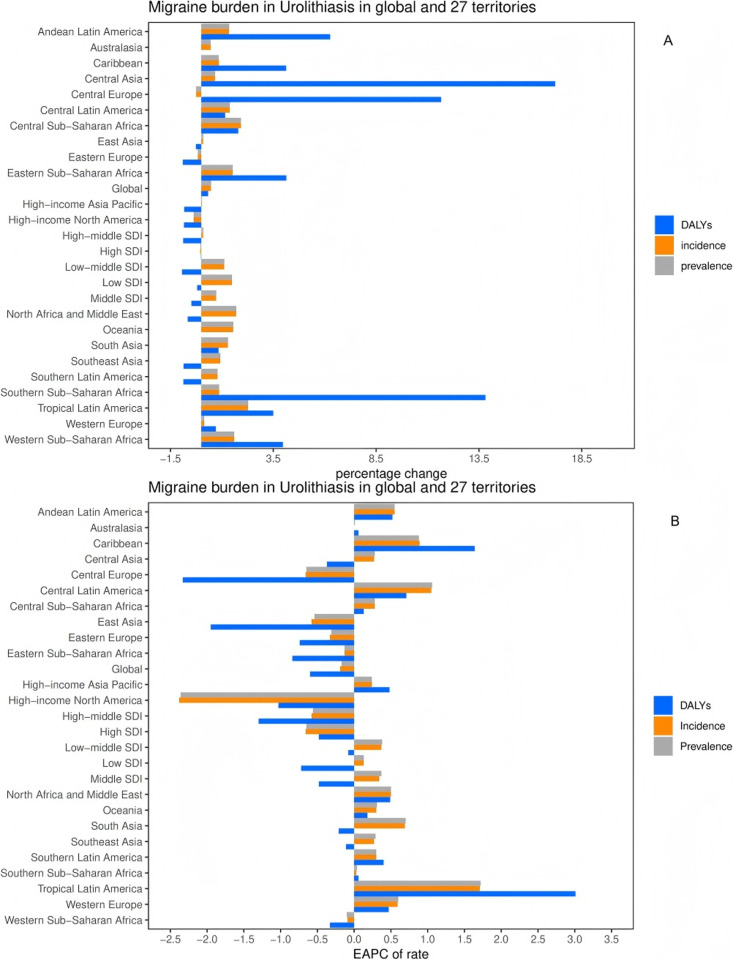
Temporal trend of migraine burden in WCBA in regions. (A) Percentage change in cases of prevalent, incident, and DALYs in 1990 and 2021. (B) EAPC of rates of prevalent, incident, and DALYs from 1990 to 2021.EAPC = Estimated Annual Percentage Change, DALYs = Disability-Adjusted Life Years.

The prevalence, incidence, and absolute numbers of DALYs of urinary stones in the 20–54 age group increased over time and were observed in most regions. Only a few regions, notably East Asia, Eastern Europe, and high-income North America, saw a decline, all of which are high SDI/ moderate-to-high SDI regions. Over the past 31 years, prevalence and disability-adjusted life years (DALYs) have continued to increase in most regions, with the greatest increases in Central Asia and southern sub-Saharan Africa; The EAPC for prevalence was 0.28 (95%CI: 0.22–0.33) and 0.04 (95%CI:-0.02–0.09), respectively, and the EAPC for DALYs was −0.37 (95%CI:-0.02–0.09), respectively. −0.53--0.21) and 0.06 (95%CI:-0.35–0.46). In contrast, in high-income North America, East Asia, and Eastern Europe, both prevalence and DALY rates have declined; The EAPC for prevalence was −2.36 (95% CI: 2.66–2.07), 0.54 (95% CI: 0.72–0.37) and 0.31 (95% CI: 0.38–0.23), the DALY EAPC respectively 1.03 (95% CI: 1.28–0.78), 1.95 (95% CI: 2.17–1.72) and 0.74 (95% CI: 0.87–0.61). The incidence and prevalence of urinary calculi in different regions showed the same trend in 20–54 age group. In East Asia, Eastern Europe, and high-income North America, there is a clear downward trend in the prevalence, incidence, and number and rate of DALYs of urinary stones in the 20–54 age group, indicating that the burden of urinary stones is decreasing in this region. This trend is likely to be closely related to improvements in health care and economic conditions (Tables 1, S1 and S2, Fig 3A and 3B). In addition to having the highest disease burden, the areas with moderate SDI are also expanding the fastest, necessitating priority action. While low SDI locations required resources to contain the danger, high SDI areas could successfully lower the burden through public health expenditure.

### National level

**Fig 4:**
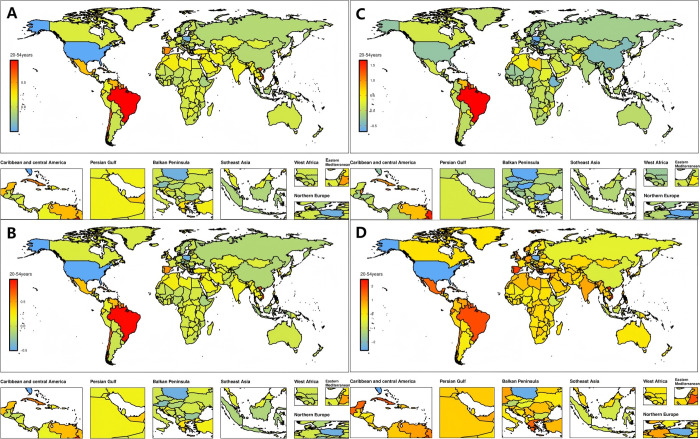
Temporal trend of migraine burden in 20-54years globally. (A) Percentage change in prevalent rate across 204 countries in 1990 and 2021. (B) Percentage change in incidence rate across 204 countries in 1990 and 2021. (C) Percentage change in DALYs rate cases across 204 countries in 1990 and 2021. (D) EAPC in prevalent rates across 204 countries from 1990 to 2021. EAPC = Estimated Annual Percentage Change.

From 1990 to 2021, the prevalence, incidence and DALYs of urinary stones among 20–54 years old have increased in about 86% of countries. The U.S. Virgin Islands, Trinidad and Tobago, and Brazil had the highest percentage change, with all three classified as moderate SDI regions, ranging from 80% to 95%. This is in sharp contrast to the significantly lower percentage changes observed in other regions with moderate SDI. In addition, only 13% of countries showed a reduction in prevalence, incidence and DALYs, such as the United States (high SDI), Poland and Australia (high SDI), with percentage reductions ranging from 39% to 41%. This suggests that the increase in urinary stone cases in the 20–54 age group is polarized between countries in high SDI regions and those in middle SDI. Over the past 32 years, prevalence rates and disability-adjusted annual rates have increased in most countries; The largest increase observed from high SDI regions was in Spain, with an EAPC of 1.9 (95% CI: 1.63–2.16), contrary to the global trend in high SDI regions. In some countries with low SDI, the incidence showed an increasing trend. Most significant increase was seen in Syrian Arab Republic, EAPC = 0.87 (95%CI: 0.74–1.01), respectively. A few countries showed decreases in incidence, with the most pronounced reductions in the United States, with Eapcs of − 2.6 (95% CI, − 2.92 to 2.27), and in Poland and Australia, with Eapcs of − 2.46 (95% CI, − 3.26 to 1.66) (Table 2 and S3, Fig 4A ~ D). More attention has to be paid to some countries’ abnormally high growth rates. Effective prevention and control led to a decrease in the burden of disease in nations with high SDI, which may highlight the critical role that economic investment and medical intervention play in reducing disease.

**Table 2:** Percentage change in prevalent, incidence, DALYs rate across 204 countries in 1990 and 2021.

location	prevalence rate (100%)			incidence rate (100%)			DALYs rate (100%)		
	1990	2021	Percentage change	1990	2021	Percentage change	1990	2021	Percentage change
Afghanistan	40.21854348	39.8225766	−0.009845381	1065.941801	1060.985663	−0.004649539	4.445796529	6.590686941	0.482453571
Albania	52.48388355	53.14677275	0.012630338	1387.147702	1403.871074	0.012055942	5.160752753	4.33862807	−0.159303249
Algeria	38.3688206	46.69002339	0.216874083	1023.353597	1240.546186	0.212236112	3.229749441	4.520367918	0.399603282
American Samoa	41.90513563	54.67702436	0.30478099	1126.534374	1462.24029	0.297998822	3.596223413	4.364169019	0.213542241
Andorra	66.65258289	78.42613416	0.176640586	1780.627608	2083.343466	0.170005147	6.201660506	6.762393999	0.09041667
Angola	28.89391501	29.76405454	0.030114975	770.3481421	794.0717207	0.030795918	3.64859147	3.391958187	−0.070337632
Antigua and Barbuda	41.99886307	54.42137273	0.295782046	1125.029148	1457.0599	0.2951308	3.967588698	6.28499267	0.584083721
Argentina	99.57139201	99.36289742	−0.002093921	2648.352821	2640.918938	−0.002806984	7.584359637	7.771430148	0.024665301
Armenia	87.65190982	116.6087306	0.33036155	2331.953466	3109.606768	0.333477196	14.16456739	12.72564964	−0.101585718
Australia	64.8522807	66.45198814	0.024666942	1733.300088	1771.34853	0.021951445	6.20139139	6.138312302	−0.010171764
Austria	151.1525015	104.0235889	−0.311797106	4066.943343	2775.677002	−0.317502909	13.16084724	9.077860991	−0.310237341
Azerbaijan	71.0241219	77.56776442	0.092132678	1884.031748	2054.295145	0.09037183	6.534869405	6.46090299	−0.011318729
Bahamas	41.93943245	55.14324287	0.314830451	1123.83068	1476.117859	0.313469979	4.754441308	8.550542814	0.79843272
Bahrain	45.02884983	56.25575804	0.249327004	1201.501747	1493.802521	0.243279524	3.575767067	4.986230259	0.394450524
Bangladesh	49.88411315	55.44515986	0.111479314	1326.705029	1472.960049	0.110239289	8.053960568	6.100234562	−0.242579535
Barbados	44.93584853	62.92962598	0.400432573	1204.106026	1688.258126	0.402084276	5.585560885	9.712389574	0.738838726
Belarus	166.0462758	182.3806738	0.098372564	4451.285077	4879.791432	0.096265763	20.7916451	22.26221231	0.070728757
Belgium	72.49945897	75.22121826	0.037541788	1934.149303	2002.597865	0.035389492	6.343763184	6.385515463	0.006581626
Belize	39.83578045	51.56142227	0.294349494	1068.326178	1385.250701	0.296655206	4.082122915	8.527155591	1.088902213
Benin	27.55177608	28.5974855	0.037954338	732.1005308	760.3143464	0.038538171	4.00297093	3.63551403	−0.091796045
Bermuda	47.24590934	62.71738116	0.327466907	1263.302073	1676.082287	0.326747041	4.38862683	6.668462165	0.519487171
Bhutan	48.70817509	55.22780635	0.133850863	1297.070722	1467.805659	0.13163117	8.344327524	6.783781893	−0.187018741
Bolivia (Plurinational State of)	83.5861334	86.99020688	0.040725337	2231.949654	2318.894291	0.038954569	8.944007539	8.598186315	−0.038665131
Bosnia and Herzegovina	53.67663816	54.6821663	0.018733069	1418.474907	1443.592629	0.017707555	6.265635816	4.933624897	−0.212589904
Botswana	32.73826021	36.45486392	0.113524777	868.6096686	965.4781468	0.111521299	3.716714066	3.842875001	0.033944213
Brazil	27.78205622	54.23390643	0.952119959	742.2219183	1444.202306	0.945782347	4.465400096	11.88526074	1.661634004
Brunei Darussalam	61.3323749	69.14241378	0.12733958	1648.593543	1850.93616	0.122736509	6.262773557	6.902402894	0.10213196
Bulgaria	72.79724754	56.40680855	−0.225151905	1942.004279	1488.835885	−0.233350873	15.95803259	6.354863559	−0.601776502
Burkina Faso	28.7589881	29.33207207	0.019927126	763.2646399	779.1513551	0.020814164	4.188461607	3.862127196	−0.077912714
Burundi	34.24139513	34.84785431	0.017711287	909.2322915	926.2633541	0.018731256	6.113476418	4.947481307	−0.19072538
Cabo Verde	26.3096341	31.63202737	0.202298263	699.9649311	839.0055261	0.198639373	2.74976423	3.144513042	0.143557331
Cambodia	51.38106252	61.03504705	0.187889935	1384.337856	1642.702993	0.186634452	9.344806083	9.133369791	−0.022626076
Cameroon	28.7581961	29.17805092	0.014599484	763.4179243	775.4351796	0.015741385	5.243936458	4.404536074	−0.160070663
Canada	45.87903576	48.71722405	0.061862422	1216.135806	1291.206889	0.061729194	4.305696835	5.015670728	0.164891752
Central African Republic	28.23181244	31.26594845	0.107472236	752.8927388	836.239588	0.110702156	4.018755739	4.209189786	0.04738632
Chad	28.21669313	28.0106903	−0.007300743	749.4748528	744.826716	−0.006201858	3.629786451	3.684612799	0.015104566
Chile	92.24935254	158.2475282	0.715432399	2459.965358	4224.29739	0.717218243	7.759731339	13.19288866	0.70017338
China	60.12042144	53.61598045	−0.108190209	1627.179123	1437.364085	−0.116652823	9.982153828	5.946545966	−0.404282275
Colombia	37.82086222	43.00921194	0.137182217	1012.676014	1147.487222	0.13312373	4.912519211	4.913387738	0.000176799
Comoros	34.64357333	39.63379876	0.144044767	919.3405934	1049.64788	0.141739947	5.77326797	6.12303157	0.060583296
Congo	28.05466469	32.30964238	0.151667387	748.2190422	861.3627513	0.151217361	4.422164707	4.415134659	−0.00158973
Cook Islands	44.30340303	53.68524314	0.211763419	1186.563236	1433.31756	0.207957163	3.586357669	4.072324351	0.135504243
Costa Rica	37.73233762	41.83833728	0.108819117	1010.871022	1116.128475	0.104125503	3.410504243	3.848061434	0.12829692
C么te d’Ivoire	28.22780646	29.92431282	0.060100538	749.9985858	794.8264102	0.059770545	3.891128622	3.928952569	0.009720559
Croatia	56.56291275	66.45234128	0.174839449	1494.402956	1765.110103	0.181147358	6.877628183	5.258944335	−0.235354952
Cuba	46.38729908	73.44822508	0.583369296	1242.646308	1975.851974	0.590035685	5.358954035	10.59143465	0.976399608
Cyprus	55.88514059	57.9434711	0.036831445	1490.640448	1542.127802	0.034540424	5.639109367	5.072702632	−0.100442587
Czechia	65.03519337	59.71163364	−0.081856599	1729.479016	1576.928941	−0.088205797	13.16744239	5.472970014	−0.584355879
Democratic People’s Republic of Korea	51.99568178	62.37636802	0.199645161	1399.969945	1680.381625	0.200298357	9.784420467	9.965706167	0.018527996
Democratic Republic of the Congo	27.33933776	30.17980614	0.103896752	728.3008079	805.1554966	0.105526024	3.115805388	3.382176554	0.085490309
Denmark	60.46205492	61.76826709	0.021603834	1610.860515	1644.672397	0.02098995	6.47975189	5.917751553	−0.086731768
Djibouti	33.8049599	39.70632695	0.174571041	898.2596876	1053.892231	0.173260078	6.041138466	7.3061221	0.209394908
Dominica	42.13533579	51.74543638	0.228076991	1128.150965	1384.725866	0.227429581	3.460635555	4.749746215	0.372506911
Dominican Republic	40.0912517	47.36628168	0.181461782	1074.591564	1267.957724	0.179943865	3.776885432	4.602051405	0.218477894
Ecuador	91.89417255	119.3484548	0.298759774	2455.918274	3190.230172	0.298996879	7.93863544	10.76866544	0.356488217
Egypt	40.97292949	44.80730635	0.093583176	1090.204957	1191.941347	0.093318591	3.820687817	4.517564062	0.182395495
El Salvador	37.12301595	38.64986088	0.041129334	993.6185147	1032.564427	0.039196041	4.121562765	4.424155838	0.073417073
Equatorial Guinea	28.23110968	28.95029749	0.02547501	752.294221	772.6937322	0.0271164	3.887100355	3.439535297	−0.115141112
Eritrea	34.79426389	37.80440482	0.086512563	922.9152361	1003.114589	0.086897853	7.527806927	7.003313597	−0.069674121
Estonia	166.3377824	179.0245537	0.076271134	4444.354458	4777.214206	0.07489496	18.87312199	17.48595169	−0.073499779
Eswatini	32.12265964	34.36660135	0.069855415	852.4508544	911.6440632	0.069438852	4.090345484	5.394077498	0.31873396
Ethiopia	40.691072	34.61907297	−0.149221899	1078.757552	919.9697448	−0.147195083	11.8536042	5.833924236	−0.507835411
Fiji	41.11072659	48.74041474	0.185588745	1102.603206	1304.390703	0.183010077	3.136091502	3.797533718	0.21091292
Finland	60.30553217	60.71932004	0.006861524	1605.467203	1615.42811	0.006204367	5.482728997	5.225509993	−0.046914411
France	65.37502682	68.54395801	0.048473115	1744.132285	1823.56647	0.04554367	5.751487074	5.757146915	0.000984066
Gabon	28.71793932	32.17143477	0.120255685	765.454691	858.5177478	0.121578792	3.644957557	4.143015367	0.136642966
Gambia	27.74648882	28.57341614	0.029802954	737.5805399	759.8739641	0.030225071	3.905496804	4.228704762	0.082757194
Georgia	69.58106302	67.65541035	−0.027674953	1842.065362	1795.603601	−0.025222646	6.138930021	6.585861963	0.072802905
Germany	61.8531313	63.61952665	0.028557897	1648.077456	1692.47988	0.026941952	5.59320726	5.400422824	−0.034467601
Ghana	29.17962381	31.45472112	0.077968699	775.4683	836.5366228	0.07875025	4.767834609	5.133316518	0.076655744
Greece	72.20496105	94.26561348	0.305528209	1924.856534	2509.741909	0.303859205	5.475578484	7.006750551	0.279636585
Greenland	42.56064696	44.55473779	0.046852926	1131.024194	1180.959507	0.044150526	3.516183688	3.5990197	0.0235585
Grenada	42.48469743	69.01120543	0.624377943	1140.056169	1861.786208	0.633065334	5.880780655	11.18190243	0.901431644
Guam	43.32526832	52.21556708	0.205198931	1164.192508	1396.034305	0.199143866	3.554517305	3.902099791	0.097786129
Guatemala	37.10522585	37.05514687	−0.001349647	993.0221557	991.1789764	−0.001856131	7.61432867	6.522227605	−0.143427098
Guinea	29.05276754	28.21857476	−0.028713023	770.9161896	750.1279396	−0.026965642	4.063311157	3.725804451	−0.083061989
Guinea-Bissau	28.71244341	28.56465287	−0.005147265	762.8160737	759.7179352	−0.004061449	6.668595749	5.263698699	−0.210673596
Guyana	41.30497747	58.44582438	0.414982599	1108.85027	1573.018993	0.418603607	5.329024013	13.73160866	1.576758638
Haiti	42.06671646	46.01599862	0.093881398	1128.564507	1237.442498	0.096474762	7.170853954	7.341623261	0.023814361
Honduras	38.97340847	37.96457636	−0.025885139	1043.793676	1017.387285	−0.025298478	12.6884536	10.66245307	−0.15967277
Hungary	68.38151646	60.03760268	−0.122020017	1822.617902	1591.924297	−0.126572665	23.99953114	7.560749775	−0.684962605
Iceland	56.11575839	60.55414993	0.079093496	1498.67539	1613.012347	0.07629201	4.731454021	5.332661704	0.127066158
India	57.82803664	67.91901722	0.174499796	1539.016547	1802.02618	0.17089461	9.832784842	8.992457817	−0.085461753
Indonesia	69.60287715	60.26937613	−0.134096483	1881.908525	1622.55859	−0.13781219	9.825995339	9.180040083	−0.065739422
Iran (Islamic Republic of)	41.77135373	49.23175151	0.178600814	1112.176096	1306.117711	0.174380312	6.321792753	5.81546534	−0.080092378
Iraq	40.09212397	45.08832674	0.124618062	1069.633681	1199.818005	0.12170926	4.203873269	4.304992573	0.024053842
Ireland	64.71546673	69.39441709	0.072300342	1727.605543	1845.906615	0.0684769	5.773408174	5.729529494	−0.007600135
Israel	61.2698637	64.21936748	0.048139552	1636.581783	1712.608232	0.046454414	5.947395734	5.91922889	−0.004735996
Italy	86.45583189	89.58408025	0.036183196	2310.825303	2394.537843	0.036226252	7.839292859	7.299513241	−0.068855652
Jamaica	40.86463917	48.70758827	0.191925079	1095.151506	1305.542886	0.192111666	4.012398148	8.493503029	1.116814612
Japan	84.5350764	87.2566115	0.032194152	2255.621468	2324.408362	0.030495761	6.758668337	7.744766917	0.145901312
Jordan	38.61538587	58.4022871	0.512409776	1030.080515	1555.587713	0.510161284	3.049449937	4.502851937	0.476611202
Kazakhstan	91.00180451	100.8650484	0.108385147	2423.637631	2697.567953	0.113024455	16.77504305	18.85062419	0.123730302
Kenya	34.49181673	38.06757907	0.103669875	916.7168164	1010.836319	0.102670204	4.557705183	5.944788392	0.304338072
Kiribati	40.42694253	44.53550791	0.101629387	1088.007618	1196.355614	0.099583857	3.742299863	3.872844504	0.034883533
Kuwait	45.52867359	51.34758172	0.127807548	1212.97611	1362.432609	0.12321471	3.413275181	4.192073553	0.228167473
Kyrgyzstan	86.033492	83.2129914	−0.032783751	2284.598919	2207.341816	−0.033816484	9.912846426	8.801405614	−0.112121258
Lao People’s Democratic Republic	56.87503073	58.29351507	0.024940371	1531.598943	1566.834223	0.023005552	10.6084072	7.834819843	−0.261451819
Latvia	170.7725605	176.5272215	0.033697808	4583.385531	4714.473381	0.02860066	23.5486248	22.64649323	−0.03830931
Lebanon	40.92933865	45.6148592	0.114478286	1087.416238	1213.173668	0.115647923	4.946631187	4.404328973	−0.109630614
Lesotho	32.79592492	33.94037745	0.034896181	868.9315156	900.4741681	0.036300505	3.3230576	5.295254842	0.593488732
Liberia	28.34851065	30.23723284	0.066625094	752.997062	803.3578581	0.066880468	4.147623789	4.286130959	0.033394343
Libya	41.3972483	48.39049821	0.168930308	1102.995159	1284.52671	0.164580551	3.482678583	5.933420263	0.703694476
Lithuania	173.8140997	175.0230425	0.006955378	4669.55289	4670.876054	0.00028336	21.42325171	21.71671704	0.01369845
Luxembourg	74.6456317	77.28463588	0.035353766	1991.769663	2057.772615	0.033137844	6.142708431	6.096559801	−0.00751275
Madagascar	34.06208668	36.61471721	0.074940521	904.0021265	971.7973569	0.074994547	5.257786195	5.03829156	−0.041746588
Malawi	33.48231501	34.40910659	0.027680033	889.5729619	914.934033	0.028509265	5.226792486	5.589169674	0.069330701
Malaysia	55.89129651	61.63496418	0.102764975	1502.513132	1654.258696	0.100994501	5.064877422	5.68674214	0.122779816
Maldives	53.18856251	72.76060472	0.367974641	1430.185869	1956.046672	0.367687036	4.169537104	5.519324714	0.32372601
Mali	29.64489597	28.4613595	−0.039923785	786.6189495	756.9204839	−0.037754577	5.009572441	3.820801297	−0.237299921
Malta	72.55625782	74.8416269	0.031497891	1936.106588	1993.345161	0.029563751	6.664849537	6.587488649	−0.011607297
Marshall Islands	39.69917204	49.08462602	0.236414351	1067.723206	1316.01509	0.232543305	3.400282983	4.114226312	0.209965856
Mauritania	29.01012305	29.58731672	0.019896285	770.6023479	786.1058039	0.02011862	5.360874408	4.046984945	−0.245088648
Mauritius	55.97965345	70.27193902	0.255312148	1505.990237	1881.424132	0.249293711	4.539134732	5.582644923	0.229891874
Mexico	52.35460062	73.47258352	0.403364416	1409.803287	1969.136961	0.3967459	10.49242554	12.32720581	0.174867123
Micronesia (Federated States of)	41.95234833	49.35889744	0.176546711	1128.088658	1325.121825	0.174661066	3.82375065	4.111375214	0.075220533
Monaco	70.43113243	71.90632666	0.020945201	1873.53035	1908.927862	0.018893482	5.35430359	5.538309693	0.03436602
Mongolia	69.83752654	75.69350833	0.083851506	1854.806321	2005.323415	0.081149764	7.128499139	6.882687466	−0.034482949
Montenegro	54.24864477	54.47320303	0.004139426	1433.495075	1438.485455	0.003481268	4.424747741	4.262779659	−0.036605043
Morocco	39.07355021	45.47177153	0.163748144	1040.674695	1208.023634	0.160808118	3.316175482	4.866782971	0.467589094
Mozambique	36.13459623	35.93722983	−0.005461979	958.2859037	954.2086874	−0.004254697	6.093733316	7.409806519	0.215971578
Myanmar	57.29599617	65.48814305	0.142979395	1543.859119	1759.203739	0.139484631	10.37387599	8.918912178	−0.14025267
Namibia	32.7804881	34.50051126	0.052470944	869.760788	914.1596363	0.051047195	3.516125575	3.792652942	0.078645475
Nauru	46.14817527	48.43311431	0.049513096	1240.103402	1300.964264	0.049077248	4.639668015	4.364472858	−0.059313545
Nepal	50.71865831	52.68677705	0.038804629	1347.486789	1400.528238	0.039363242	7.616834286	6.566067241	−0.13795325
Netherlands	67.04146729	69.75626426	0.040494295	1790.937538	1857.49443	0.037163157	7.078431589	6.811865796	−0.037658878
New Zealand	71.23558735	67.27456877	−0.055604491	1914.779195	1794.951256	−0.062580552	8.214009221	7.229124041	−0.119903101
Nicaragua	35.63527826	39.03684689	0.095455088	955.2288108	1043.754056	0.092674388	4.155244809	4.524898488	0.088960746
Niger	28.69594455	27.72858432	−0.033710695	762.073199	737.7043945	−0.031976987	3.918483401	3.137823962	−0.199224894
Nigeria	31.41262214	29.34183993	−0.065921979	833.4667105	779.1226599	−0.065202425	3.934489016	3.453983105	−0.122126637
Niue	47.58997576	55.71113067	0.170648435	1274.955664	1489.49913	0.168275236	3.914190989	4.370699209	0.11662901
North Macedonia	53.98176387	54.64261584	0.012242134	1425.847618	1442.703399	0.011821586	4.360897752	4.165656147	−0.044770966
Northern Mariana Islands	44.76921822	58.09642284	0.297686784	1200.996753	1550.312202	0.290854615	3.623947021	4.353870309	0.201416655
Norway	112.6750703	117.8814135	0.0462067	3017.018762	3147.297466	0.043181271	9.102739165	9.101252533	−0.000163317
Oman	47.57143915	53.76618969	0.130219952	1268.155347	1431.45608	0.128770291	3.685086667	4.33996586	0.177710662
Pakistan	54.43413042	53.65220714	−0.014364577	1447.778637	1426.557229	−0.014657909	8.825411645	9.599796069	0.08774485
Palau	43.13529015	64.35867145	0.492018976	1158.523938	1717.961357	0.482888096	3.723534144	5.109335669	0.372173712
Palestine	36.71087114	41.97246274	0.143325163	980.0907976	1118.459551	0.141179525	3.089561514	3.380411182	0.094139465
Panama	38.5588015	41.75553305	0.082905366	1031.488012	1113.445354	0.079455448	4.195260715	4.457897838	0.06260329
Papua New Guinea	40.36109036	43.55040031	0.07901942	1083.334801	1167.49669	0.077687792	3.245628802	3.366628041	0.037280677
Paraguay	40.00645756	41.21992586	0.030331811	1065.531771	1097.305142	0.029819262	3.833731664	5.673185367	0.47980763
Peru	85.67717131	92.51102381	0.079762817	2288.948532	2463.073886	0.076072202	7.332507715	7.829213602	0.067740247
Philippines	92.10947492	108.3345759	0.176150184	2488.255124	2913.405103	0.170862696	17.73309124	20.69901675	0.167253722
Poland	69.41088809	39.04640589	−0.437459929	1853.619863	1029.132813	−0.444798347	11.84908443	3.877717965	−0.672741131
Portugal	57.20723997	69.9143391	0.222123968	1524.081318	1863.378998	0.222624394	5.483153231	5.853469716	0.06753714
Puerto Rico	46.58592485	54.92377763	0.178977938	1245.530896	1467.968915	0.178588921	4.144850269	7.571277177	0.826670853
Qatar	51.87525818	58.62053448	0.130028775	1381.996704	1559.986181	0.128791535	4.340515095	4.627783802	0.066183091
Republic of Korea	63.20357947	80.12148644	0.267673241	1693.177842	2132.996539	0.259759304	5.254435029	6.289290758	0.196949001
Republic of Moldova	151.5526666	168.1513024	0.109523878	4052.515796	4488.62593	0.107614666	16.43211114	18.28526954	0.112776647
Romania	55.8716897	57.70414168	0.032797504	1476.459032	1523.675795	0.031979731	4.252495434	4.316759275	0.01511203
Russian Federation	200.7863189	178.2320393	−0.112329763	5399.890317	4765.511931	−0.117479865	26.70671615	21.87316307	−0.180986425
Rwanda	34.09089352	35.98255344	0.055488717	904.6261113	955.9400571	0.056723927	5.998578097	4.624446643	−0.229076196
Saint Kitts and Nevis	39.51791737	56.42757769	0.42789857	1059.950211	1511.528285	0.426037062	3.714274386	5.305879273	0.428510315
Saint Lucia	40.71572575	60.25138924	0.479806343	1091.616144	1612.123257	0.476822476	5.006991724	10.18660809	1.034476718
Saint Vincent and the Grenadines	41.04617234	64.18790759	0.563797644	1101.285134	1724.519986	0.565915977	5.07007967	8.629813256	0.702106045
Samoa	40.4939468	47.87763796	0.182340615	1088.687316	1283.254686	0.178717403	3.356555333	3.866654958	0.151971165
San Marino	65.3680718	73.89973657	0.130517308	1743.375958	1961.832502	0.125306618	5.51470884	6.098336316	0.105831059
Sao Tome and Principe	28.21922878	31.36123237	0.111342646	749.5648446	832.4218333	0.110540121	4.163067539	4.569327522	0.09758669
Saudi Arabia	44.19001058	52.14820074	0.180090252	1177.488146	1385.883701	0.176983145	3.33110903	4.009792845	0.203741099
Senegal	28.11210199	29.04215331	0.033083663	746.872523	771.756751	0.033317905	4.311159557	3.943574588	−0.085263596
Serbia	54.95864012	54.09931662	−0.015635822	1451.091818	1428.158975	−0.015803854	5.056446396	4.561165399	−0.09795041
Seychelles	54.49594806	78.02592936	0.431774877	1466.426782	2091.8068	0.426465218	5.442145411	8.635776391	0.586833085
Sierra Leone	28.05291967	28.51851025	0.016596867	745.1880954	758.2635067	0.017546457	3.54984224	3.611612163	0.017400752
Singapore	64.05672727	80.10034541	0.250459535	1719.425307	2136.139184	0.242356487	5.372209908	6.595134626	0.227639042
Slovakia	62.12510188	62.07741432	−0.000767605	1642.898803	1639.59198	−0.002012797	7.449935856	5.418033224	−0.272740957
Slovenia	52.47415612	54.03031977	0.029655811	1385.724625	1426.087739	0.029127803	6.760963821	4.434124219	−0.344157973
Solomon Islands	42.98779135	50.2415615	0.168740238	1155.242433	1347.312887	0.166259868	3.617096679	4.116995109	0.138204332
Somalia	36.66949058	37.45281319	0.021361699	973.6868593	997.0309911	0.023974989	8.443973277	7.832707609	−0.072390763
South Africa	36.70346051	37.16780683	0.012651295	973.1120915	983.4996605	0.010674586	4.433506471	4.119844056	−0.070748158
South Sudan	32.43713766	36.20601928	0.116190327	862.3845806	961.4198161	0.114838829	6.153759854	7.393245243	0.2014192
Spain	57.0745409	88.32575976	0.547550946	1522.024125	2342.821095	0.539279869	5.310948872	7.19780194	0.355276075
Sri Lanka	58.1867462	67.20022609	0.154906065	1563.98441	1799.430296	0.150542348	4.923295159	5.596434595	0.136725387
Sudan	38.5256263	40.33694665	0.047015987	1026.188137	1075.17885	0.047740479	3.385648575	4.399255017	0.299383241
Suriname	43.55324361	69.92980073	0.605616366	1168.733049	1893.299787	0.619959142	5.44395068	10.72541032	0.970152
Sweden	71.10415018	62.92451688	−0.115037354	1897.707509	1679.589292	−0.114937742	6.344295322	5.185795725	−0.182604929
Switzerland	72.25691966	74.94012508	0.037134235	1927.44251	1994.322921	0.034699043	6.069901571	5.979668938	−0.014865584
Syrian Arab Republic	38.82983776	48.21881018	0.241797879	1036.050864	1278.028329	0.233557514	4.343664194	4.758406377	0.095482101
Taiwan (Province of China)	47.63197442	74.57167198	0.565580115	1281.950973	2001.865818	0.561577517	5.232390696	7.569459314	0.446654073
Tajikistan	72.55581479	78.43412861	0.081017818	1926.49601	2078.83476	0.079075559	11.20995694	9.484532977	−0.153918875
Thailand	70.88364859	82.2992149	0.161046539	1916.451304	2207.261843	0.151744288	16.27176281	15.32378788	−0.058258896
Timor-Leste	52.45229418	53.42952388	0.018630828	1411.013363	1436.272742	0.017901588	6.711675657	6.288613372	−0.06303378
Togo	27.60034838	29.90382883	0.083458383	733.735946	794.1440717	0.082329516	4.09557063	4.373639952	0.067895135
Tokelau	42.44885973	50.95939823	0.200489214	1139.006513	1363.385953	0.196995748	3.625742356	4.079412671	0.125124808
Tonga	40.52213223	47.10650634	0.162488343	1088.011558	1263.143929	0.160965543	3.288375672	3.72328836	0.132257604
Trinidad and Tobago	48.93189438	94.46597909	0.930560431	1317.900677	2580.309945	0.957894089	10.92401919	27.54553346	1.521556671
Tunisia	39.45408432	47.98636608	0.216258517	1050.94726	1273.164731	0.211444931	3.176285432	4.372110647	0.376485439
T眉rkiye	40.78850178	48.67618345	0.193380029	1085.615177	1293.216814	0.191229491	4.376245686	4.3999352	0.005413205
Turkmenistan	69.17569546	77.08700695	0.114365478	1836.01438	2044.3766	0.113486159	6.39579441	7.58541186	0.185999951
Tuvalu	43.42413766	48.81071612	0.124045721	1165.662409	1308.73577	0.122739962	3.896839709	3.941534542	0.011469508
Uganda	32.09652675	33.55142132	0.045328723	852.5142524	892.2043872	0.046556565	4.32498951	4.771367579	0.103209052
Ukraine	202.3377859	204.1609286	0.009010392	5456.396241	5474.29369	0.003280086	21.04431209	19.00975891	−0.096679482
United Arab Emirates	50.96047727	69.80710655	0.369828351	1357.501382	1847.501385	0.360957278	4.086004492	5.359776021	0.31174012
United Kingdom	77.19285178	91.77862993	0.188952446	2061.707967	2445.73879	0.186268293	7.161949432	8.897317611	0.242303886
United Republic of Tanzania	33.07579998	35.90521829	0.085543458	878.1338881	953.3016257	0.085599404	5.717559876	5.371588493	−0.060510321
United States of America	89.34459266	44.67776435	−0.499938798	2382.968573	1184.240857	−0.503039667	7.542666497	5.284633712	−0.299367974
United States Virgin Islands	57.72600474	103.912036	0.800090557	1548.892784	2826.640355	0.824942555	10.92428542	12.92862584	0.183475655
Uruguay	100.6663733	102.4224638	0.017444659	2675.456054	2718.811926	0.01620504	8.338424497	9.813387491	0.176887492
Uzbekistan	70.45913901	75.80882038	0.075926011	1870.644148	2009.762992	0.074369486	5.243773677	5.634312396	0.074476654
Vanuatu	40.39242513	44.72044647	0.107149331	1084.425019	1199.191914	0.105832024	3.30929819	3.566839007	0.077823394
Venezuela (Bolivarian Republic of)	39.0150887	55.64976736	0.426365266	1045.48954	1485.361609	0.420733113	6.246315163	11.12454896	0.780977852
Viet Nam	54.23409094	92.46298294	0.704886748	1461.943914	2479.014361	0.695697309	5.216992761	8.10388306	0.553362911
Yemen	39.18689031	41.02915745	0.047012333	1043.756671	1093.367023	0.047530572	3.255259947	4.098195997	0.258945849
Zambia	35.01036908	37.6237028	0.074644564	929.1776504	999.9039587	0.0761171	6.671448706	7.276963541	0.090762121
Zimbabwe	33.36746206	35.28587289	0.05749346	884.0001448	931.4965104	0.053728912	3.217794929	4.436098984	0.378614574

### Age and sex patterns

**Fig 5:**
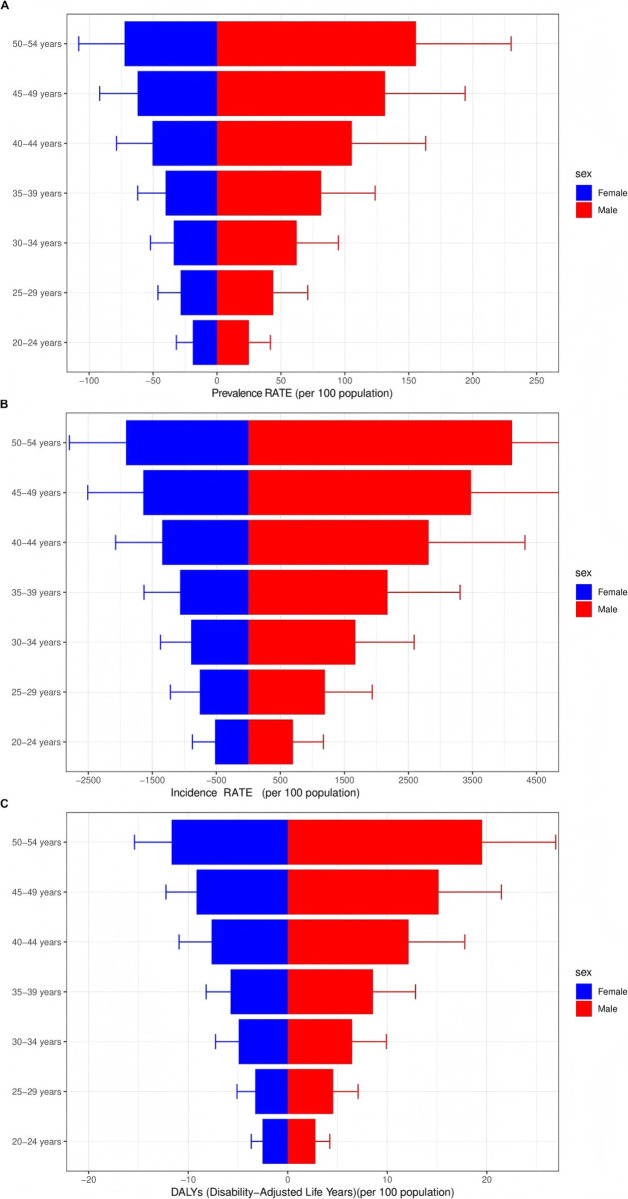
Sex- and age-structured analysis of urolithiasis disease burden in 2021. (A) Prevalence rates; **(B)** Incidence rates; **(C)** DALYs rates.

In 2021, the global prevalence of Urolithiasis peaked in the 50–54 age bracket within the 20–54 age range and subsequently declined with advancing age. Across all ages, men exhibited a higher prevalence than women (Fig 5A; Supplementary S4 table). Regarding new Urolithiasis cases in 2021, the incidence was notably higher among men (2178 cases) compared to women (1115 cases) (Supplementary S8 Table). Specifically, the highest incidence occurred in the 50–54 age group, closely followed by the 45–49 and 40–44 age groups. Notably, significant gender disparities were observed in each age group, with men consistently having higher incidence rates than women. It is worth mentioning that the incidence rate among men aged 20–24 increased more rapidly than that among women in the same age group, and the largest gender gap was observed in the 50–54 age group (4118 men vs. 1910 women) (Fig 5B; Supplementary S5 Table). Analysis of Disability-Adjusted Life Years (DALYs) rates revealed similar trends to prevalence and incidence, with DALYs rates escalating with age. However, the sex-related DALYs rate was most comparable in the 20–24 age group (men: 2.78, women: 2.54), whereas the widest sex-related DALYs rate disparities were found in the 50–54 age group (men: 19.53, women: 11.67) and the 45–49 age group (men: 15.15, women: 9.16) (Fig 5C; Supplementary S6 Table). Interestingly, in every age group, men had greater disease burdens than women. This cohort revealed the narrowest gender disparity in DALY rates (male 2.78 vs female 2.54), indicating that gerden-related risk factors may worsen with age, even though males aged 20–24 years showed faster incidence rise than females of the same age. Men between the ages of 50 and 54 had the highest burden of urolithiasis, which calls for focused preventative measures.

### Overall temporal trends in gender and age structures

**Fig 6:**
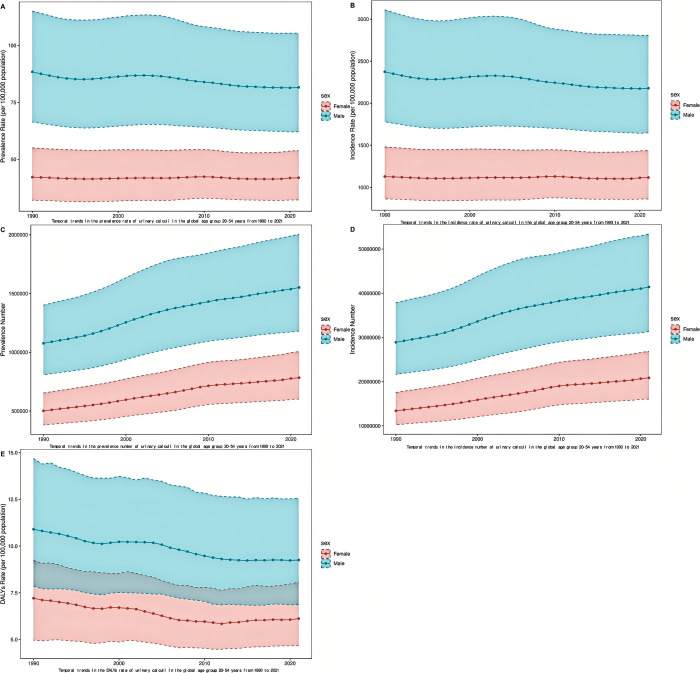
Global temporal trends in urolithiasis disease burden, 1990–2021. (A) Prevalence rates in 20-54 age groups. (B) Incidence rates in 20-54 age groups. (C) Prevalence number; (D) Incidence number; (E) DALYs rate.

From 1990 to 2021, the prevalence and incidence of urolithiasis within all age groups ranging from 20 to 54 years old generally fluctuated insignificantly. Specifically, throughout this period, the prevalence and incidence rates among males were consistently much higher than those among females. Despite a notable increase being observed during the period from 1995 to 2005, and a distinct downward trend emerging from 2005 to 2021, the overall prevalence and incidence ultimately demonstrated a downward trend (Fig 6A-B; Supplementary S7 and S8 tables). Conversely, the number of individuals affected and the number of cases in the 20–54 age group increased significantly, with the most pronounced increase occurring in the interim from 2000 to 2005 (Fig 6C-D). Among the 20–54 age group, the overall variations in DALYs were similar to those in prevalence. It is notable that DALYs for males remained higher than those for females (Fig 6E; Supplementary S9 Table). Gender differences in the burden of urolithiasis have existed for a long time, and the contradiction between the increase in the absolute number of urolithiasis cases and the decrease in the age-standardized rate is mainly due to the change in population structure

### Temporal joinpoint analysis

**Fig 7:**
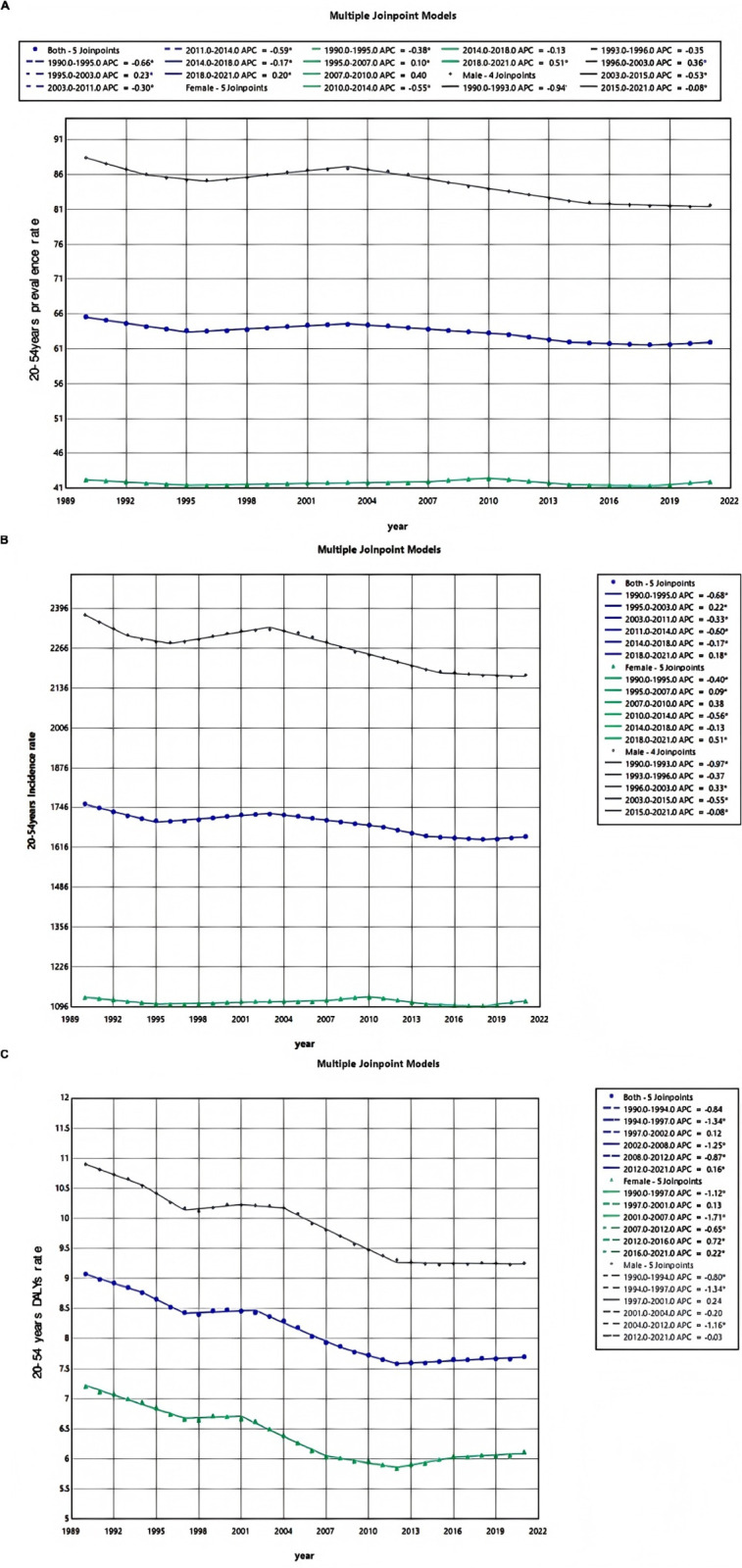
Joinpoint regression analysis of the urolithiasis disease burden temporal trends, 1990–2021. (A) 20-54years prevalence rates; (B) 20-54years incidence rates; (C) 20-54years DALYs rates.

Joint point regression analysis revealed that from 1990 to 2021, the prevalence of urolithiasis among individuals aged 20–54 years exhibited a significant overall downward trend (AAPC = −18.39%; 95% CI: −21.01% to −15.77%). Notably, there was an upward trend observed from 1995 to 2003 (APC = 23.2%; 95% CI: 20.34%−26.06%; P < 0.001) (Fig 7A and Supplementary S10 Table). Similarly, the incidence of urolithiasis in this age group also demonstrated a marked overall decline (AAPC = −20.11%; 95% CI: −22.74% to −17.48%; P < 0.001), with the most pronounced reductions occurring between 1990–1995 and during the period of 2011–2014 (APC = −68.03%; 95% CI: −72.88% to −63.19%; P < 0.001 and APC = −59.52%; 95% CI: −79.58% to −39.42%), while a notable increase in incidence was recorded from 1995 to 2003 (APC = 21.51%; 95% CI: 18.61% to 24.40%) (Fig 7B and Supplementary S11 Table). Changes in male incidence significantly contributed to these trends.Disability-adjusted life years (DALYs) serve as a crucial metric for assessing health burden, providing substantial support for public health policy formulation and medical research by quantifying health loss attributable to disease or injury.From 1990 to 2021, there was an overall significant decrease in DALYs among individuals aged 20–54 years due to urolithiasis (AAPC = −53.06%; 95% CI: −62.06% to −44.05%; P < 0.001). The most significant declines were noted between 1990 and 1994 and from 2008 to 2012 (APC = −84.50%; 95% CI: −106.43% to −62.51%, P < 0.001 and APC = −87.48%; 95% CI: −121.66% to −53.18%). Importantly, DALYs in females continued to rise between 2012 and 2021 (APC = 72.13%; 95% CI: 26.39% to 118.07%, and APC = 21.58%; 95% CI: 1.16% to 42.05%), while a gradual decline was observed among males (Fig 7C and Supplementary S12 Table). The global prevalence, incidence, and DALYs of urolithiasis among individuals aged 20–54 declined dramatically between 1990 and 2021, but there were sporadic variations. The years 1995–2003 had a modest spike in both prevalence and incidence, while 1990–1994 and 2008–2012 saw the largest declines in DALYs. However, from 2012 to 2021, female DALYs increased more, suggesting that the gender gap was getting worse.

### The association between urolithiasis burden and SDI

**Fig 8:**
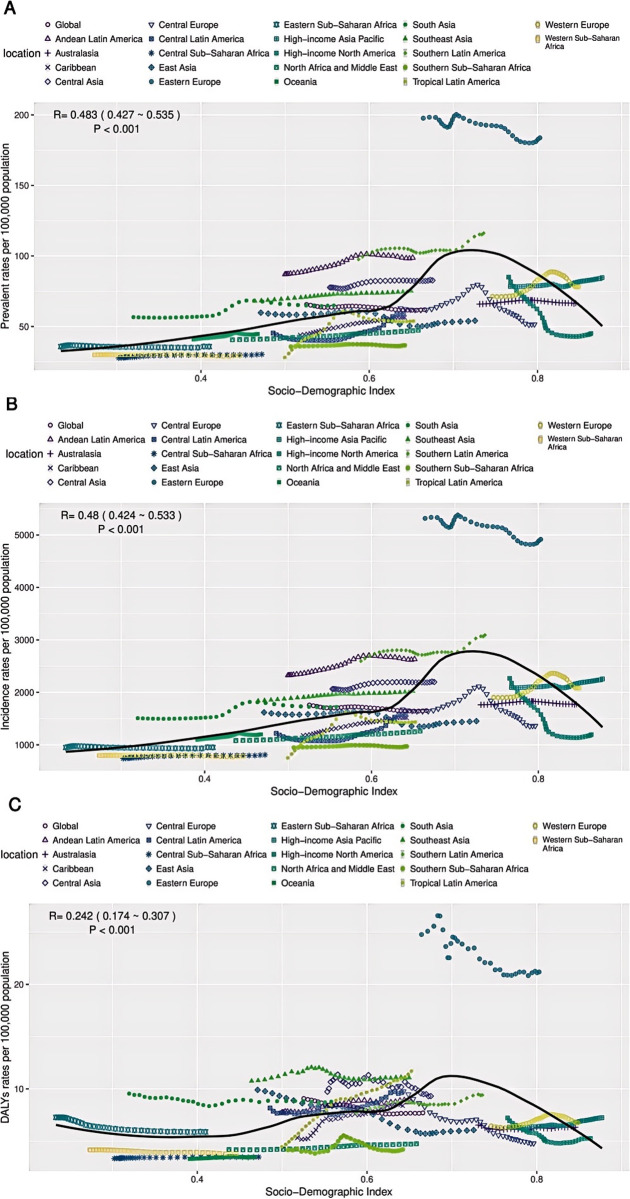
The associations between the SDI and prevalent, incidence, DALYs rates per 100,000 population of migraine in 20-54years across 21 GBD regions. SDI = Socio-Demographic Index, GBD = Global Burden of Disease (A) prevalent (B) inceidence **(C)** DALYs.

In 2021, there was a positive correlation between the prevalence, incidence, and DALY rates of urolithiasis in the 20–54 age group and the Sociodemographic Index (SDI). The overall burden of disease is increasing as the economy improves. In 21 regions, the most significant rise in urolithiasis burden was observed in the 20–54 age group when SDI values ranged from 0.6 to 0.7. The burden of urolithiasis peaked when the SDI reached 0.7. However, when the SDI value exceeded 0.7, the prevalence and incidence of urolithiasis were negatively correlated with the DALY rates in the 20–54 age group. Notably, Eastern European countries exhibited significantly higher prevalence and DALY rates compared to other regions (Fig 8A–C). The greatest rise in illness burden was found in 21 regions with SDIs between 0.6 and 0.7, which are primarily moderate. Regional disparities in prevention and control as well as unequal access to medical resources may be the reason why the prevalence and DALY rate in Eastern European nations were substantially higher than those in other regions.

## Discussion

As part of the new Agenda 2030, the United Nations set the Sustainable Development Goals (SDGs) in 2015 with the aim of “promoting physical and mental health and well-being, extending life expectancy for all individuals, and ensuring universal health coverage and access to quality healthcare.” By 2030, the ultimate objective is to lower mortality and reduce the burden of disease on the world’s population [20]. The worldwide society is becoming aware that urinary tract disorders pose a serious danger to global health. Urolithiasis, in particular, is becoming increasingly common, posing significant difficulties to global public health policies and healthcare systems. A recent assessment of epidemiological data from seven nations found that kidney stone incidence rates ranged from 114 to 720 per 100,000 people, with prevalence rates ranging from 1.7% to 14.8%. Notably, these rates appear to be increasing in almost all investigated countries [21]. According to statistics from the National Health and Nutrition Examination Survey (NHANES), the prevalence of self-reported kidney stones in the United States than quadrupled, rising from 3.2% in 1976–1980 to 8.8% in 2007–2010 [22,23]. Furthermore, from 2000 to 2010, the lifetime prevalence of kidney stones in the United Kingdom increased by 63%, from 7.14% to 11.62% [24]. The aim of this study is to conduct a comprehensive analysis of global trends and patterns in the prevalence, incidence, and disability-adjusted life years (DALYs) associated with urolithiasis for individuals aged 20–54. Our findings indicate that the overall worldwide burden, measured in terms of both the total number of cases and the population afflicted, has grown since 1990, which is consistent with current literature across all age categories [25].

However, it is critical to recognize that these trends range across countries, gender classifications, and geographical regions.

This study seeks to provide a thorough assessment of the estimated burden and trends related to urolithiasis among people aged 20–54 at the national, regional, and worldwide levels from 1990 to 2021 using data from the Global Burden of Disease (GBD) 2021. According to a number of studies, working-age persons have a significantly higher frequency of stone disease, while older populations have a lower incidence [13,26,27]. According to a French study, the highest prevalence of urolithiasis was seen in women aged 30–39, whereas the highest incidence occurred in men aged 40–49. According to calculations, the overall male-to-female (M/F) ratio was 2.28 [27]. Corresponding data from an epidemiological examination in Germany that used telephone interviews, the prevalence of urolithiasis was 9.7% in males and 5.9% in women over the age of 5 [28]. A cohort study in Korea showed that the highest incidence of urolithiasis occurred at ages 50–54 years for men and 55–59 years for women [29]. Multiple studies have found that the occurrence of stones has increased significantly among both men and women aged 25 and up, with men showing a particularly noticeable tendency. Furthermore, a study of the age distribution of stone patients between 1979 and 2001 demonstrated that the observed rise in prevalence and incidence was primarily due to the occurrence of stones in the older age group (>50 years). Conversely, there is also evidence indicating that an increasing number of young women are now affected by urinary calculi [28]. Undoubtedly, a comprehensive understanding of urinary stone prevalence trends in the 20–54 age group is essential to assess the potential to achieve related health goals. However, a comprehensive analysis of the prevalence, incidence and DALYs of urinary stones in this population in different countries and regions of the world is lacking. Therefore, we believe that there is a need for timely strengthening and updating of the global data on urinary stone burden in the 20–54 age group so that policy makers can be informed and develop effective prevention and control strategies. The study is the first to provide a comprehensive estimate of the prevalence, incidence and DALYs of urinary stones in patients aged 20–54 years over the past 32 years using GBD 2021 data on a global scale. Over the past 32 years, there has been a notable rise in the incidence of urinary stones, the number of reported cases, and the associated disability-adjusted life years (DALYs) within the global 20–54 age group, with percentage increases of 48%, 47%, and 33%, respectively. This uptick may be correlated with a 45% increase in the global population. Conversely, the overall prevalence, DALY rate, and incidence of urinary stones have been declining on a global scale over time. These findings suggest that while the number of patients has increased, the rate of new case emergence is decelerating. This trend can be attributed to major advances in diagnosis, prevention, and prognosis from the clinical experience accumulated by urological professionals with physicians over the past 32 years, as well as the development of advanced diagnostic and therapeutic technical systems. In 2021, low-SDI areas exhibited the lowest rates of prevalence, incidence, and DALYs, while medium-high SDI areas reported the highest figures. However, both prevalence and incidence in low-SDI regions increased by 150% from 1990 to 2021. This trend aligns with previous epidemiological transition models, which indicated a greater burden of urolithiasis in the 20–54 age group in countries with higher SDI levels [30]. Higher valuesof the Social Development Index (SDI) are often associatedwith stronger healthcare systems and higher-quality medical services, both of which lead to a lower disease burden. In this investigation, the highest prevalence, incidence, and disability-adjusted life years (DALYs) associated with urolithiasis in the 20–54 age group were observed in regions classified as moderately high SDI, while the most substantial rise in cases was documented in low SDI regions.This study implies that countries with moderate to high SDI frequently have regions with more sophisticated economies and better social infrastructure. Countries with enormous populations, such as China and India, have a proportionally big number of patients. Furthermore, as the economies of middle and high SDI regionsgrow, so does the number of reported causes and diagnoses of urolithiasis among those aged 20–54.However, it is important to recognize that growing urbanization and industrialization have caused significant shifts in living choices. These alterations include increased sedentary behavior, higher professional stress, a lack of physical activity due to overexertion, and insufficient access to water or restroom facilities which impedes regular hydration [31]. Such conditions may cause decreased fluid intake and urine output, increasing the risk of stone formation and worsening the incidence of urolithiasis [32,33]. Certain vocations that require prolonged exposure to high temperatures, such as steelworkers, glass producers, and mechanics, have an increased risk of acquiring urinary stones [34]. In a study of Swedish battery industry workers, cumulative cadmium exposure was linked to an increase the risk of kidney stone formation [35]. A 7-year prospective analysis found that long-term cadmium exposure is related with an increased risk of upper urinary tract stones [36]. Furthermore, a case study revealed that the risk of kidney stones among drivers could be linked to tight workplace requirements surrounding toilet breaks [37]. The Korean study’s postulated “healthy worker effect” is a significant problem in epidemiological studies on occupational health. Employed people had a higher prevalence and incidence of urinary stones than unemployed people [38]. The rise in the frequency, incidence, and disability-adjusted life years (DALYs) associated with urolithiasis among the 20–54 age group in low SDI nations is especially noticeable. This may be attributed to advancements in medical facilities, such as the widespread use of computers, driven by economic development and international support. Tomography (CT) and X-rays in clinical practice [39], as well as increased awareness of self-care in the population.As a result, urolithiasis detection rates have increased significantly. According to research, patients in wealthy locations and those with private insurance are more likely to receive imaging diagnosis, such as CT scans, during medical appointments. Patients from developing countries, on the other hand, are less likely to undergo any type of diagnostic imaging, such as KUB, ultrasound, or CT [40]. Urinary stone prevalence and incidence appear to be higher in developing nations with rapid socioeconomic growth. Although various elements may be involved, improvements in socioeconomic conditions are obviously important [25]. By encouraging environmental governance policies to lower the risk of heavy metal and mineral pollution, strengthening urban and rural infrastructure to ensure clean drinking water supply, improving the basic medical security system to cover stone screening and treatment, publicizing health education to raise awareness of disease prevention, improving the industrial structure to reduce agricultural and industrial pollution, and improving residents’ employment and income to improve diet and medical access.

In terms of age and gender pattern, the prevalence and incidence of urinary stones in the global 20–54 age range increased steadily with age in 2021. Urinary stone prevalence and incidence were substantially lower in the 20–24 age group compared to the 50–54 age group.This trend may be related to the metabolism of calcium oxalate, the most prevalent component of kidney stones. However, there are significant regional differences: in Europe, the incidence of calcium oxalate stones rises between the ages of 40 and 50, whereas in North Africa, it peaks between 16 and 39 years [41]. In contrast, Wu et al. found that the peak age for calcium oxalate stones in southern China is between 19 and 40 years [42]. The researchers Yang et al., on the other hand, found that the peak prevalence occurred in eastern China between the ages of 30 and 50 [43]. Various ethnic groups, regions, and food habits all contribute to this. Hypertension and metabolic syndrome have both been linked to the development of kidney stones in southern Italian populations [44]. In contrast, in South Korea, the odds ratio for the presence of metabolic syndrome among 34,895 individuals undergoing general health screening was shown to be 1.25 in connection to the occurrence of kidney stones [45]. So it’s more common in older people than in younger people in the same age group.

Males appear to be more susceptible to urolithiasis than females, with prior research indicating a male-to-female ratio ranging from 1.7:1–3:1 [46,47]. Furthermore, calcium oxalate and uric acid stones are more common observed in men than in women, which is consistent with current research in specific regions of China [42,48]. The actual pathophysiology driving the sex difference in urolithiasis is unknown, while various putative causes have been discovered. In terms of eating habits, males consume more alcohol and coffee and eat more meat than females [49]. Physiologically, testosterone can promote stone formation, but estrogen appears to decrease it via controlling the production of 1,25-dihydroxyvitamin D [50]. However, the conclusions of numerous investigations appear to disagree. For example, some studies emphasize the inhibitory effect of estrogen on stone formation, while others imply that estrogen replacement therapy in postmenopausal women may be a risk factor for urolithiasis [51].

In summary, our study found that the global burden of urolithiasis among people aged 20–54 reduced between 1990 and 2021. However, given the importance of this age group for social productivity, urolithiasis is anticipated to continue causing large health and developmental costs, particularly among the 50–54 age group in middle-and high-SDI countries like China and India. It is critical to emphasize that urolithiasis itself carries significant health hazards. Urolithiasis patients had a 1.3 times higher chance of developing diabetes, 1.5 times higher risk of hypertension, twice the risk of metabolic syndrome, and a 2–4 times higher risk of cardiovascular disease [52]. These dangers necessitate additional preventive policies. Furthermore, while developing global health targets, disparities in the burden of urolithiasis across genders and age groups should be taken into account in order to develop more effective and suitable medical and health policies for urolithiasis prevention and early treatment.

The limitations of this study begin with the constraints of the GBD study itself. The primary data sources include censuses, household surveys, civil registration and vital statistics, satellite imaging,and so on. As a result, the quality of the data gathered in this study varied, and the predictive value of the modeling effort was obtained without the original data. Furthermore, the GBD study made no distinction between urolithiasis kinds, which may differ by geography. Stone compositio and location are not distinguished, which makes it impossible to guide prevention strategies, and the over-inclusion of asymptomatic stones overestimates the actual medical needs.As a result, the quality of the data gathered in this study varied, and the predictive value of the modeling effort was determined without the original data. Furthermore, gender differences may be influenced by clinic bias, and the “high burden” of SDI stratification may reflect high diagnostic rates rather than true incidence. As a result, more research is needed to explore the prevalence patterns of urolithiasis in different sections of the urogenital tract and with various chemical compositions. In the future, multi-center prospective cohort studies are needed to integrate stone composition analysis, environmental exposure monitoring and health economic evaluation to develop precise prevention and control strategies.

## Conclusions

In conclusion, since 1990, there has been an increase in urolithiasis cases, DALYs, and fatalities worldwide among those aged 20–54. While the frequency and DALY rates in low SDI countries grew at the quickest rate, those in high SDI countries were declining. Men had a greater prevalence rate and DALY rate than women did. Because of the enormous illness burden that urolithiasis causes, policies for particular age groups must be developed in order to prevent and treat urolithiasis, alleviate the social medical and health burden, and lower the working age group’s quality of life and productivity.

## Supporting information

S1 Table The incidence of urolithiasis cases and rates among 20-54 in 1990 and 2021, and the trends from 1990 to 2021. (XLSX)

S2 Table The DALY of urolithiasis cases and rates among 20-54 in 1990 and 2021, and the trends from 1990 to 2021. (XLSX)

S3 Table Percentage change in EAPC. (XLSX)

S4 Table 20-54 Structure of urolithiasis Prevalence Rate among Males and Females, 2021. (XLSX)

S5 Table 20-54 Structure of urolithiasis Incidence Rate among Males and Females, 2021. (XLSX)

S6 Table 20-54 Structure of urolithiasis DALYs Rate among Males and Females, 2021. (XLSX)

S7 Table 20-54 and Sex Structure of urolithiasis Prevalence, 1990-2021. (XLSX)

S8 Table 20-54 and Sex Structure of urolithiasis Incidence, 1990-2021. (XLSX)

S9 Table 20-54 and Sex Structure of urolithiasis DALYs, 1990-2021. (XLSX)

S10 Table Temporal Joinpoint Analysis of urolithiasis 20-54 Prevalence Rates, 1990-2021. (XLSX)

S11 Table Temporal Joinpoint Analysis of urolithiasis 20-54 Incidence Rates, 1990-2021. (XLSX)

S12 Table Temporal Joinpoint Analysis of urolithiasis 20-54 DALYs Rates, 1990-2021. (XLSX)

## References

[1] Sorokin I, Mamoulakis C, Miyazawa K, Rodgers A, Talati J, Lotan Y. Epidemiology of stone disease across the world. World J Urol. 2017;35(9):1301–20. doi: 10.1007/s00345-017-2008-6 28213860

[2] Ramello A, Vitale C, Marangella M. Epidemiology of nephrolithiasis. J Nephrol. 2000;13(Suppl 3):S45–50. 11132032

[3] Pinduli I, Spivacow R, del Valle E, Vidal S, Negri AL, Previgliano H, et al. Prevalence of urolithiasis in the autonomous city of Buenos Aires, Argentina. Urol Res. 2006;34(1):8–11. doi: 10.1007/s00240-005-0003-7 16425020

[4] Medina-Escobedo M, Zaidi M, Real-de León E, Orozco-Rivadeneyra S. Urolithiasis prevalence and risk factors in Yucatan, Mexico. Salud Publica Mex. 2002;44(6):541–5. 20383456

[5] Trinchieri A. Epidemiology of urolithiasis: an update. Clin Cases Miner Bone Metab. 2008;5(2):101–6. 22460989 PMC2781200

[6] Baştuğ F, Gündüz Z, Tülpar S, Poyrazoğlu H, Düşünsel R. Urolithiasis in infants: evaluation of risk factors. World J Urol. 2013;31(5):1117–22. doi: 10.1007/s00345-012-0828-y 22258667

[7] Shin S, Srivastava A, Alli NA, Bandyopadhyay BC. Confounding risk factors and preventative measures driving nephrolithiasis global makeup. World J Nephrol. 2018;7(7):129–42. doi: 10.5527/wjn.v7.i7.129 30510912 PMC6259033

[8] Knoll T. Epidemiology, pathogenesis, and pathophysiology of urolithiasis. Eur Urol Suppl. 2010;9(12):802–6.

[9] Hyams ES, Matlaga BR. Economic impact of urinary stones. Transl Androl Urol. 2014;3(3):278–83. doi: 10.3978/j.issn.2223-4683.2014.07.02 26816777 PMC4708578

[10] Antonelli JA, Maalouf NM, Pearle MS, Lotan Y. Use of the National Health and Nutrition Examination Survey to calculate the impact of obesity and diabetes on cost and prevalence of urolithiasis in 2030. Eur Urol. 2014;66(4):724–9. doi: 10.1016/j.eururo.2014.06.036 25015037 PMC4227394

[11] Saigal CS, Joyce G, Timilsina AR, Urologic Diseases in America Project. Direct and indirect costs of nephrolithiasis in an employed population: opportunity for disease management?. Kidney Int. 2005;68(4):1808–14. doi: 10.1111/j.1523-1755.2005.00599.x 16164658

[12] Hiatt RA, Dales LG, Friedman GD, Hunkeler EM. Frequency of urolithiasis in a prepaid medical care program. Am J Epidemiol. 1982;115(2):255–65. doi: 10.1093/oxfordjournals.aje.a113297 7058784

[13] Knoll T, Schubert AB, Fahlenkamp D, Leusmann DB, Wendt-Nordahl G, Schubert G. Urolithiasis through the ages: data on more than 200,000 urinary stone analyses. J Urol. 2011;185(4):1304–11. doi: 10.1016/j.juro.2010.11.073 21334658

[14] GBD 2019 Diseases and Injuries Collaborators. Global burden of 369 diseases and injuries in 204 countries and territories, 1990-2019: a systematic analysis for the Global Burden of Disease Study 2019. Lancet. 2020;396(10258):1204–22. 33069326 10.1016/S0140-6736(20)30925-9 PMC7567026

[15] Global incidence, prevalence, years lived with disability (YLDs), disability-adjusted life-years (DALYs), and healthy life expectancy (HALE) for 371 diseases and injuries in 204 countries and territories and 811 subnational locations, 1990-2021: a systematic analysis for the Global Burden of Disease Study 2021. Lancet. 2024;403(10440). 10.1016/S0140-6736(24)00757-8 PMC11122111 38642570

[16] Yang X, Zhang T, Zhang X, Chu C, Sang S. Global burden of lung cancer attributable to ambient fine particulate matter pollution in 204 countries and territories, 1990-2019. Environ Res. 2022;204(Pt A):112023. doi: 10.1016/j.envres.2021.112023 34520750

[17] Yang X, Chen H, Zhang T, Yin X, Man J, He Q, et al. Global, regional, and national burden of blindness and vision loss due to common eye diseases along with its attributable risk factors from 1990 to 2019: a systematic analysis from the global burden of disease study 2019. Aging (Albany NY). 2021;13(15):19614–42. doi: 10.18632/aging.203374 34371482 PMC8386528

[18] Zhang L, Tong Z, Han R, Li K, Zhang X, Yuan R. Spatiotemporal trends in global burden of rheumatic heart disease and associated risk factors from 1990 to 2019. Int J Cardiol. 2023;384:100–6. doi: 10.1016/j.ijcard.2023.04.060 37149003

[19] Joinpoint Regression Program [EB/OL]. https://surveillance.cancer.gov/joinpoint/. Accessed 2024 October 9

[20] Transforming our world: the 2030 Agenda for Sustainable Development (A/RES/70/1)[EB/OL]. https://refugeesmigrants.un.org/transforming-our-world-2030-agenda-sustainable-development-ares701. 2015. Accessed 2024 October 13

[21] Romero V, Akpinar H, Assimos DG. Kidney stones: a global picture of prevalence, incidence, and associated risk factors. Rev Urol. 2010;12(2–3):e86–96. 20811557 PMC2931286

[22] Stamatelou KK, Francis ME, Jones CA. Time trends in reported prevalence of kidney stones in the United States: 1976-1994. Kidney Int. 2003;63(5):1817–23. 12675858 10.1046/j.1523-1755.2003.00917.x

[23] Scales CD Jr, Smith AC, Hanley JM, Saigal CS, Urologic Diseases in America Project. Prevalence of kidney stones in the United States. Eur Urol. 2012;62(1):160–5. doi: 10.1016/j.eururo.2012.03.052 22498635 PMC3362665

[24] Turney BW, Reynard JM, Noble JG, Keoghane SR. Trends in urological stone disease. BJU Int. 2012;109(7):1082–7. doi: 10.1111/j.1464-410X.2011.10495.x 21883851

[25] Alatab S, Pourmand G, El Howairis MEF, Buchholz N, Najafi I, Pourmand MR, et al. National profiles of urinary calculi: A comparison between developing and developed worlds. Iran J Kidney Dis. 2016;10(2):51–61. 26921745

[26] Croppi E, Ferraro PM, Taddei L, Gambaro G, GEA Firenze Study Group. Prevalence of renal stones in an Italian urban population: a general practice-based study. Urol Res. 2012;40(5):517–22. doi: 10.1007/s00240-012-0477-z 22534684

[27] Daudon M, Doré J-C, Jungers P, Lacour B. Changes in stone composition according to age and gender of patients: a multivariate epidemiological approach. Urol Res. 2004;32(3):241–7. doi: 10.1007/s00240-004-0421-y 15127165

[28] Trinchieri A, Coppi F, Montanari E, Del Nero A, Zanetti G, Pisani E. Increase in the prevalence of symptomatic upper urinary tract stones during the last ten years. Eur Urol. 2000;37(1):23–5. doi: 10.1159/000020094 10671780

[29] Study on the prevalence and incidence of urolithiasis in Korea over the last 10 years: An analysis of National Health Insurance Data PubMed[EB/OL]. https://pubmed.ncbi.nlm.nih.gov/30402571/. Accessed 2024 October 13 10.4111/icu.2018.59.6.383 PMC6215783 30402571

[30] Omran AR. The epidemiologic transition: a theory of the epidemiology of population change. 1971. Milbank Q. 2005;83(4):731–57. doi: 10.1111/j.1468-0009.2005.00398.x 16279965 PMC2690264

[31] Y L, Y C, B L, et al. Epidemiology of urolithiasis in Asia. Asian J Urol. 2018;5(4). 10.1016/j.ajur.2018.08.007 PMC6197415 30364478

[32] Curhan GC. Epidemiology of stone disease. Urol Clin North Am. 2007;34(3):287–93. doi: 10.1016/j.ucl.2007.04.003 17678980 PMC2693870

[33] Goldfarb DS, Arowojolu O. Metabolic evaluation of first-time and recurrent stone formers. Urol Clin North Am. 2013;40(1):13–20. doi: 10.1016/j.ucl.2012.09.007 23177631 PMC4052537

[34] Atan L, Andreoni C, Ortiz V. High kidney stone risk in men working in steel industry at hot temperatures. Urology. 2005;65(5):858–61. 15882711 10.1016/j.urology.2004.11.048

[35] Järup L, Elinder CG. Incidence of renal stones among cadmium exposed battery workers. Br J Ind Med. 1993;50(7):598–602. doi: 10.1136/oem.50.7.598 8343420 PMC1035495

[36] Scott R, Cunningham C, McLelland A, Fell GS, Fitzgerald-Finch OP, McKellar N. The importance of cadmium as a factor in calcified upper urinary tract stone disease--a prospective 7-year study. Br J Urol. 1982;54(6):584–9. doi: 10.1111/j.1464-410x.1982.tb13601.x 6758912

[37] Lee W, Kang M-Y, Kim J, Lim S-S, Yoon J-H. Cancer risk in road transportation workers: a national representative cohort study with 600,000 person-years of follow-up. Sci Rep. 2020;10(1):11331. doi: 10.1038/s41598-020-68242-5 32647239 PMC7347601

[38] Heo J, Son J, Lee W. Epidemiology of urolithiasis with sex and working status stratification based on the national representative cohort in Republic of Korea. Saf Health Work. 2022;13(4):482–6. doi: 10.1016/j.shaw.2022.07.002 36579016 PMC9772479

[39] Gadzhiev N, Prosyannikov M, Malkhasyan V, Akopyan G, Somani B, Sivkov A, et al. Urolithiasis prevalence in the Russian Federation: analysis of trends over a 15-year period. World J Urol. 2021;39(10):3939–44. doi: 10.1007/s00345-021-03729-y 34008087

[40] Schoenfeld D, Mohn L, Agalliu I, Stern JM. Disparities in care among patients presenting to the emergency department for urinary stone disease. Urolithiasis. 2020;48(3):217–25. doi: 10.1007/s00240-019-01136-y 31025079

[41] Wang S, Zhang Y, Zhang X, Tang Y, Li J. Upper urinary tract stone compositions: the role of age and gender. Int Braz J Urol. 2020;46(1):70–80. doi: 10.1590/S1677-5538.IBJU.2019.0278 31851461 PMC6968895

[42] W W, B Y, L O. Urinary stone analysis on 12,846 patients: a report from a single center in China. Urolithiasis. 2014;42(1). 10.1007/s00240-013-0633-0 24362574

[43] Yang X, Zhang C, Qi S. Multivariate analyses of urinary calculi composition: a 13-year single-center study. J Clin Lab Anal. 2016;30(6):873–9. 27075109 10.1002/jcla.21950 PMC6807181

[44] Rendina D, Mossetti G, De Filippo G, Benvenuto D, Vivona CL, Imbroinise A, et al. Association between metabolic syndrome and nephrolithiasis in an inpatient population in southern Italy: role of gender, hypertension and abdominal obesity. Nephrol Dial Transplant. 2009;24(3):900–6. doi: 10.1093/ndt/gfn548 18835844

[45] Jeong IG, Kang T, Bang JK. Association between metabolic syndrome and the presence of kidney stones in a screened population. Am J Kidney Dis. 2011;58(3):383–8. 21620546 10.1053/j.ajkd.2011.03.021

[46] Edvardsson VO, Indridason OS, Haraldsson G, Kjartansson O, Palsson R. Temporal trends in the incidence of kidney stone disease. Kidney Int. 2013;83(1):146–52. doi: 10.1038/ki.2012.320 22992468

[47] Stamatelou KK, Francis ME, Jones CA. Time trends in reported prevalence of kidney stones in the United States: 1976-1994. Kidney Int. 2003;63(5):1817–23. 12675858 10.1046/j.1523-1755.2003.00917.x

[48] Yang X, Zhang C, Qi S. Multivariate analyses of urinary calculi composition: A 13-year single-center study. J Clin Lab Anal. 2016;30(6):873–9. 27075109 10.1002/jcla.21950 PMC6807181

[49] Zeng G, Mai Z, Xia S, Wang Z, Zhang K, Wang L, et al. Prevalence of kidney stones in China: an ultrasonography based cross-sectional study. BJU Int. 2017;120(1):109–16. doi: 10.1111/bju.13828 28236332

[50] Inter-annual variability of urolithiasis epidemic from semi-arid part of Deccan volcanic province, India: climatic and hydrogeochemical perspectives. PubMed [EB/OL]. 2024. https://pubmed.ncbi.nlm.nih.gov/23869912/10.1080/09603123.2013.818105 23869912

[51] Maalouf NM, Sato AH, Welch BJ, Howard BV, Cochrane BB, Sakhaee K, et al. Postmenopausal hormone use and the risk of nephrolithiasis: results from the Women’s Health Initiative hormone therapy trials. Arch Intern Med. 2010;170(18):1678–85. doi: 10.1001/archinternmed.2010.342 20937929 PMC3293452

[52] Lang J, Narendrula A, El-Zawahry A, Sindhwani P, Ekwenna O. Global trends in incidence and burden of urolithiasis from 1990 to 2019: An analysis of global burden of disease study data. Eur Urol Open Sci. 2022;35:37–46. doi: 10.1016/j.euros.2021.10.008 35024630 PMC8738898

