# Supplementary material for: Epidemiological trends of urolithiasis in working-age populations: Findings from the global burden of disease study 1990–2021
Source: PLoS One. 2025 Jul 1;20(7):e0327343. doi: 10.1371/journal.pone.0327343 (PMC12212516; doi:10.1371/journal.pone.0327343)
Supplement: Attachment — Submitted filename: PONE-D-24-54570.docx [file pone.0327343.s013.docx]

**Peer Review of Manuscript PONE-D-24-54570**

The manuscript titled "Urolithiasis panorama in working-age people: results from the analysis of the Global Burden of Disease database" investigates an important global health issue, providing a comprehensive analysis of urolithiasis trends from 1990 to 2021 using a robust dataset. The study is valuable for understanding the burden of this condition in working-age populations and offers insights that could inform public health strategies.

While the manuscript is well-structured and the methodology is statistically rigorous, there are significant areas that require improvement. Specifically, the clarity and accessibility of the text, the depth of the discussion, and the practical implications of the findings need refinement. This review identifies the weaknesses, and actionable suggestions to improve the manuscript for acceptance.

**Abstract**

**Weaknesses:**

1. **Overuse of technical terms:** While the abstract includes key findings, it may not be easily understood by a broader audience due to terms like "EAPC," "SDI," and "DALYs" being introduced without explanation.
2. **Lack of focus on implications:** While findings are well-presented, the abstract could better emphasize the practical implications of the results (e.g., healthcare strategies or policy recommendations).
3. **No mention of limitations:** Abstracts should briefly mention significant limitations, especially for studies analyzing large datasets.

**Suggestions:**

1. Simplify technical language for accessibility.
2. Highlight actionable insights and public health implications.
3. Briefly mention the limitations of the study.

**Introduction**

**Weaknesses:**

1. **Limited context:** While the introduction describes urolithiasis prevalence, it doesn’t sufficiently address why the 20–54 age group was chosen as the focus or the gaps in prior studies.
2. **Weak research justification:** The rationale for the study is mentioned but not strongly emphasized.
3. **Inconsistent citation style:** Some claims lack proper citation, while others reference multiple sources for the same point.

**Suggestions:**

1. Expand the context to explain why focusing on the working-age group is critical.
2. Strengthen the justification by clearly outlining the gaps in prior research.
3. Ensure all claims are supported by accurate citations.

**Methods**

**Weaknesses:**

1. **Insufficient details:** While the methods describe data sources and analysis techniques, they lack details about how the data was processed (e.g., criteria for excluding outliers or handling missing data).
2. **Clarity of statistical methods:** Terms like "joinpoint regression" and "EAPC" are introduced without explanation, making it difficult for readers unfamiliar with these concepts to follow.
3. **Reproducibility issues:** The section doesn’t mention whether code or analysis scripts are available for reproducibility.

**Suggestions:**

1. Provide more detail on how data were cleaned and processed.
2. Include brief explanations or references for complex statistical methods.
3. Mention whether analysis scripts are available for reproducibility.

**Results**

**Weaknesses:**

1. **Dense presentation:** Results are presented in a dense format with little interpretation, making it difficult for readers to extract key messages.
2. **Limited focus on practical insights:** While the numbers are well-detailed, there’s insufficient focus on what these trends mean in a practical context.
3. **Underutilization of visuals:** Figures and tables are used but could be more impactful if tailored to highlight key trends and findings.

**Suggestions:**

1. Summarize key results in simpler terms to highlight major findings.
2. Provide more interpretation of what the trends indicate in terms of public health or policy.
3. Reorganize or enhance visuals (e.g., adding comparative trends or highlighting regional differences).

**Discussion**

**Weaknesses:**

1. **Lack of critical analysis:** While results are discussed, there’s limited critical engagement with potential biases or alternative explanations for the findings.
2. **Overgeneralization of results:** Some conclusions, such as the need for "focused intervention techniques," are too broad without specifying actionable recommendations.
3. **Insufficient limitations:** The study acknowledges data limitations but doesn’t address other potential weaknesses, such as reliance on global datasets or differences in healthcare systems.

**Suggestions:**

1. Critically analyze the results, including potential biases or confounding factors.
2. Provide specific recommendations for interventions or policy changes.
3. Expand the limitations section to address gaps in data and methodology.

**Recommendation: Accept with Major Modifications**

**Reasons for Acceptance:**

1. **Relevance of the Topic:** The study addresses an important global health issue (urolithiasis) and provides valuable insights into its trends across various demographic and socioeconomic groups.
2. **Robust Dataset:** The use of the Global Burden of Disease (GBD) database ensures comprehensive and reliable data.
3. **Statistical Rigor:** Advanced methods like joinpoint regression and EAPC provide nuanced analysis of trends.

**Reasons for Requiring Major Modifications:**

1. **Clarity and Accessibility:**
   - The manuscript relies heavily on unexplained technical terms, making it less accessible to a broader audience.
   - Dense writing and data presentation hinder the clear communication of findings.
2. **Discussion Weaknesses:**
   - The discussion lacks depth in addressing biases, alternative explanations, and the practical implications of the findings.
3. **Incomplete Methods Section:**
   - The methodology needs more detail, especially regarding data cleaning and handling of uncertainties.
4. **Limited Practical Recommendations:**
   - The study’s conclusions are broad and lack actionable or specific recommendations for public health interventions.

**Conditions for Acceptance**

1. **Revise Abstract:**
   - Simplify language and focus on the implications of findings for healthcare systems and policies.
2. **Strengthen Introduction:**
   - Provide a clearer rationale for focusing on the 20–54 age group and identify gaps in previous research.
3. **Expand Methods:**
   - Include more details on data cleaning, outlier handling, and reproducibility of the statistical analysis.
4. **Improve Results Presentation:**
   - Reorganize results for clarity, focus on key findings, and enhance the visual impact of figures and tables.
5. **Deepen Discussion:**
   - Critically analyze the findings, acknowledge limitations more thoroughly, and suggest specific interventions or policy changes.
6. **Polish References:**
   - Ensure all claims are cited and references align with journal guidelines.
