# Supplementary material for: Epidemiological trends of urolithiasis in working-age populations: Findings from the global burden of disease study 1990–2021
Source: PLoS One. 2025 Jul 1;20(7):e0327343. doi: 10.1371/journal.pone.0327343 (PMC12212516; doi:10.1371/journal.pone.0327343)
Supplement: Attachment — Submitted filename: Review comments.docx [file pone.0327343.s015.docx]

**Review report**

Review report of the manuscript titled **″Urolithiasis panorama in working-age people: results from the analysis of Global Burden of Disease database 1990-2021″** is below. Here are my suggestions:

**Title**

- The title is informative and reflects the study objective.

**Abstract**

- The ethical consideration is not specified.

**Introduction**

- The study rationale and objectives are clearly identified.

**Methods**

- The duration of the study is clearly specifying.
- Ethical considerations clearly identified.

**Results**

- The results highlight the study objectives.

**Discussion**

- The impact of the study on the literature is clarified.
- The findings are consistently discussed.

**References**

- The references updated and related to the study objectives.

*Best regards*
