# Supplementary material for: Epidemiological trends of urolithiasis in working-age populations: Findings from the global burden of disease study 1990–2021
Source: PLoS One. 2025 Jul 1;20(7):e0327343. doi: 10.1371/journal.pone.0327343 (PMC12212516; doi:10.1371/journal.pone.0327343)
Supplement: Attachment — Submitted filename: Response to Reviewers.docx [file pone.0327343.s016.docx]

**Response to Reviewers**

Response to Reviewers#1

**Abstract**

1. **Overuse of technical terms:** While the abstract includes key findings, it may not be easily understood by a broader audience due to terms like "EAPC," "SDI," and "DALYs" being introduced without explanation.

**Re：**We appreciate your insightful feedback. Regarding your observation the that abstract introduces terms like "EAPC", "SDI" and "DALYs" without explanation, which might not be easily understood by a wider audience, we have explained them when we first mentioned in the following text. However, due to the limited number of words in the abstract, we may not be able to elaborate on them in the abstract.

2. **Lack of focus on implications:** While findings are well-presented, the abstract could better emphasize the practical implications of the results (e.g., healthcare strategies or policy recommendations).

**Re：**Thank you for your review and feedback. In response to the questions you raised, I have added some practical significance of the research results in the abstract, which is in line 48-51.

3. **No mention of limitations:** Abstracts should briefly mention significant limitations, especially for studies analyzing large datasets.

**Re：**Thank you for your outstanding review comments, I have suggested the limitations of this study in the abstract at lines 52-53.

**Introduction**

1. **Limited context:** While the introduction describes urolithiasis prevalence, it doesn’t sufficiently address why the 20–54 age group was chosen as the focus or the gaps in prior studies.

**Re：**Thank you for your valuable advice. We have made additional comments on the following two aspects (the revised content has been marked in lines 83-91 of the manuscript).

2. **Weak research justification:** The rationale for the study is mentioned but not strongly emphasized.

**Re：**Thank you for your valuable advice. We have made additional comments on the following two aspects (the revised content has been marked in lines 92-95 of the manuscript).

3. **Inconsistent citation style:** Some claims lack proper citation, while others reference multiple sources for the same point.

**Re：**Thank you for your careful review of the rigor of literature citation. We fully agree that literature citation should follow the principle of necessity and have systematically optimized the citation.

**Methods**

1. **Insufficient details:** While the methods describe data sources and analysis techniques, they lack details about how the data was processed (e.g., criteria for excluding outliers or handling missing data).

**Re：**I thank the reviewer for his attention to my data processing methods. Regarding the data quality control standards, the following is added:All analyses and graphical presentation were performed with the use of statistical software R, version 3.5.2. Data GBD 2021, which provides the latest epidemiological data estimates of the burden of 371 diseases and injuries in 21 GBD areas and 204 countries and territories for the period 1990 to 2021. After comprehensive verification, no outliers were found in this data set, the completeness reached 100%, and no data missing occurred.

2. **Clarity of statistical methods:** Terms like "joinpoint regression" and "EAPC" are introduced without explanation, making it difficult for readers unfamiliar with these concepts to follow.

**Re：**Thanks to the reviewers for their review comments, we have included short explanations after terms like "Joinpoint regression" and "EAPC" for better understanding by readers.

3. **Reproducibility issues:** The section doesn’t mention whether code or analysis scripts are available for reproducibility.

**Re：**Thank you for your review. The following are partial codes used for the analysis of prevalence, incidence, DALYS, and the codes used for EAPC calculations.

library(dplyr)

library(ggplot2)

setwd("F:\\GBD\\20-54")

options(scipen = 200)

WCBA<-read.csv("worldIHME-GBD_2021_DATA-48862eba-1.csv")

region_order <- read.csv("order.csv",header = F)

WCBA$location <- factor(WCBA$location,

levels=region_order$V1,

ordered=TRUE)

#-------------------------Table1:患病率prevalence------------------------

##-------Number-----------

###----1990------

WCBA_1990<-subset(WCBA,

WCBA$year==1990 &

WCBA$age=='20-54 years' &

WCBA$metric=='Number' &

WCBA$measure=='DALYs (Disability-Adjusted Life Years)')

WCBA_1990<-WCBA_1990[,c(2,8,9,10)] #只需要选择需要的变量：地区及对应的数值

WCBA_1990$val<-round(WCBA_1990$val/1,2)

WCBA_1990$upper<-round(WCBA_1990$upper/1,2)

WCBA_1990$lower<-round(WCBA_1990$lower/1,2)#取两位小数

WCBA_1990$Num_1990 <- paste0("(", WCBA_1990$lower, "-", WCBA_1990$upper, ")")

WCBA_1990$Num_1990 <- paste0(WCBA_1990$val, " ", WCBA_1990$Num_1990)

###----2021------

WCBA_2021<-subset(WCBA,

WCBA$year==2021 &

WCBA$age=='20-54 years' &

WCBA$metric=='Number' &

WCBA$measure=='DALYs (Disability-Adjusted Life Years)'

)

WCBA_2021<-WCBA_2021[,c(2,8,9,10)] #只需要选择需要的变量：地区及对应的数值

WCBA_2021$val<-round(WCBA_2021$val/1,2)

WCBA_2021$upper<-round(WCBA_2021$upper/1,2)

WCBA_2021$lower<-round(WCBA_2021$lower/1,2)#取整

WCBA_2021$Num_2021 <- paste0("(", WCBA_2021$lower, "-", WCBA_2021$upper, ")")

WCBA_2021$Num_2021 <- paste0(WCBA_2021$val, " ", WCBA_2021$Num_2021)

###-----percentage change------

WCBA_1990to2021_Numchange <- cbind(WCBA_1990,WCBA_2021)

WCBA_1990to2021_Numchange$percentage_change_val <- round(((WCBA_2021$val - WCBA_1990$val)/WCBA_1990$val),2)

WCBA_1990to2021_Numchange$percentage_change_lower <- round(((WCBA_2021$lower - WCBA_1990$lower)/WCBA_1990$lower),2)

WCBA_1990to2021_Numchange$percentage_change_upper <- round(((WCBA_2021$upper - WCBA_1990$upper)/WCBA_1990$upper),2)

WCBA_1990to2021_Numchange$percentage_change <- paste0("(", WCBA_1990to2021_Numchange$percentage_change_lower, "-", WCBA_1990to2021_Numchange$percentage_change_upper, ")")

WCBA_1990to2021_Numchange$percentage_change <- paste0(WCBA_1990to2021_Numchange$percentage_change_val, " ", WCBA_1990to2021_Numchange$percentage_change)

#整理

WCBA_NUM_1990<- WCBA_1990[,c(1,5)]

WCBA_NUM_2021<- WCBA_2021[,c(1,5)]

WCBA_NUM_1990to2021 <-WCBA_1990to2021_Numchange[,c(1,11)]

WCBA_Prelavence_NUM_Table1 <-merge(WCBA_NUM_1990,WCBA_NUM_2021,by="location",all.x=T)

WCBA_Prelavence_NUM_Table1 <-merge(WCBA_Prelavence_NUM_Table1,WCBA_NUM_1990to2021,by="location",all.x=T)

##-------Rate-----------

###----1990------

WCBA_1990<-subset(WCBA,

WCBA$year==1990 &

WCBA$age=='20-54 years' &

WCBA$metric=='Rate' &

WCBA$measure=='DALYs (Disability-Adjusted Life Years)'

)

WCBA_1990<-WCBA_1990[,c(2,8,9,10)] #只需要选择需要的变量：地区及对应的数值

WCBA_1990$val<-round(WCBA_1990$val,2)

WCBA_1990$upper<-round(WCBA_1990$upper,2)

WCBA_1990$lower<-round(WCBA_1990$lower,2)#取2位小数

WCBA_1990$Rate_1990 <- paste0("(", WCBA_1990$lower, "-", WCBA_1990$upper, ")")

WCBA_1990$Rate_1990 <- paste0(WCBA_1990$val, " ", WCBA_1990$Rate_1990)

###----2021------

WCBA_2021<-subset(WCBA,

WCBA$year==2021 &

WCBA$age=='20-54 years' &

WCBA$metric=='Rate' &

WCBA$measure=='DALYs (Disability-Adjusted Life Years)')

WCBA_2021<-WCBA_2021[,c(2,8,9,10)] #只需要选择需要的变量：地区及对应的数值

WCBA_2021$val<-round(WCBA_2021$val,2)

WCBA_2021$upper<-round(WCBA_2021$upper,2)

WCBA_2021$lower<-round(WCBA_2021$lower,2)#取整

WCBA_2021$Rate_2021 <- paste0("(", WCBA_2021$lower, "-", WCBA_2021$upper, ")")

WCBA_2021$Rate_2021 <- paste0(WCBA_2021$val, " ", WCBA_2021$Rate_2021)

###-------EAPC--------

EAPC<-subset(WCBA,

WCBA$age=='20-54 years' &

WCBA$metric=='Rate' &

WCBA$measure=='Incidence')

EAPC <-EAPC[,c(2,7,8)]

country<-WCBA_1990$location

EAPC_cal <-data.frame(location = country,

EAPC = rep(0, times = 204),

UCI = rep(0, times = 204),

LCI = rep(0, times = 204))

for(i in 1:204){

country_cal <- as.character(EAPC_cal[i,1])

a <- subset(EAPC,EAPC$location==country_cal)

a$y <-log(a$val)

\# 使用lm函数进行线性回归分析

regression_model <- lm(y ~ year, data = a)

\# 计算EAPC值

eapc_value <- 100*(exp(summary(regression_model)[["coefficients"]][2,1])-1)

low_eapc <- 100*(exp(summary(regression_model)[["coefficients"]][2,1]-1.96*summary(regression_model)[["coefficients"]][2,2])-1)

high_eapc <- 100*(exp(summary(regression_model)[["coefficients"]][2,1]+1.96*summary(regression_model)[["coefficients"]][2,2])-1)

EAPC_cal[i,2] <-round(eapc_value,2)

EAPC_cal[i,3] <-round(high_eapc,2)

EAPC_cal[i,4] <-round(low_eapc,2)

}

EAPC_cal$EAPC_ci <- paste0("(", EAPC_cal$LCI, "-", EAPC_cal$UCI, ")")

EAPC_cal$EAPC_cal <- paste0(EAPC_cal$EAPC, " ", EAPC_cal$EAPC_ci)

#整理

WCBA_Rate_1990<- WCBA_1990[,c(1,5)]

WCBA_Rate_2021<- WCBA_2021[,c(1,5)]

WCBA_Rate_1990to2021_EAPC <-EAPC_cal[,c(1,6)]

WCBA_Prelavence_Rate_Table1 <-merge(WCBA_Rate_1990,WCBA_Rate_2021,by="location",all.x=T)

WCBA_Prelavence_Rate_Table1 <-merge(WCBA_Prelavence_Rate_Table1,WCBA_Rate_1990to2021_EAPC,by="location",all.x=T)

#合并

WCBA_Prelavence_Table1<-merge(WCBA_Prelavence_NUM_Table1,WCBA_Prelavence_Rate_Table1,by="location",all.x=T)

write.csv(WCBA_Prelavence_Table1,"F:\\GBD\\20-54\\1.csv")

Here are some of the codes used to analyze the prevalence, incidence, DALYs and changes of urinary stones from 1990 to 2021 by country worldwide:

library(ggmap)

library(maps)

library(dplyr)

setwd("F:\\GBD\\20-54\\worldmap")

WCBA <- read.csv("IHME-GBD_2021_DATA-48862eba-1.csv",header = T) ##??ȡ????

options(scipen = 200)

unique(WCBA$location)

#-------------------------??????revalence------------------------

###----2021------

case_2021<-subset(WCBA,WCBA$year==1990 &

WCBA$age=='20-54 years' &

WCBA$metric=='Rate' &

WCBA$measure=='Incidence'

)

case_2021$val<-case_2021$val/1

case_2021 <- case_2021[,c(2,8)]

names(case_2021) <- c('location','case_2021','upper','lower')

country_asr <- case_2021

country_asr$val <- (case_2021$case_2021) ### ??ȡ?????

#########################################################################################

\#### map

worldData <- map_data('world')

worldData <-subset(worldData,region !='Antarctica')

unique(country_asr$location)

unique(worldData$region)

country_asr$location <- as.character(country_asr$location)

##write.csv(country_asr,"F:\\GBD\\20-54\\worldmap\\2021DALYs.csv")

###???´???==????????һ???

country_asr$location[country_asr$location == 'Bolivarian Republic of Venezuela'] = 'Venezuela'

country_asr$location[country_asr$location == 'Principality of Andorra'] = 'Andorra'

country_asr$location[country_asr$location == 'Republic of the Union of Myanmar'] = 'Myanmar'

country_asr$location[country_asr$location == 'Kingdom of Belgium'] = 'Belgium'

country_asr$location[country_asr$location == 'Russian Federation'] = 'Russia'

country_asr$location[country_asr$location == 'Republic of Paraguay'] = 'Paraguay'

country_asr$location[country_asr$location == 'Islamic Republic of Afghanistan'] = 'Afghanistan'

country_asr$location[country_asr$location == 'Federative Republic of Brazil'] = 'Brazil'

country_asr$location[country_asr$location == 'Republic of Italy'] = 'Italy'

#country_asr$location[country_asr$location == 'Bolivia (Plurinational State of)'] = 'Bolivia'

country_asr$location[country_asr$location == 'Bolivia (Plurinational State of)'] = 'Bolivia'

country_asr$location[country_asr$location == 'Kingdom of Bhutan'] = 'Bhutan'

country_asr$location[country_asr$location == 'Grand Duchy of Luxembourg'] = 'Luxembourg'

country_asr$location[country_asr$location == 'State of Libya'] = 'Libya'

country_asr$location[country_asr$location == 'Republic of the Philippines'] = 'Philippines'

country_asr$location[country_asr$location == 'Republic of Vanuatu'] = 'Vanuatu'

country_asr$location[country_asr$location == 'Togolese Republic'] = 'Togo'

country_asr$location[country_asr$location == 'Eastern Republic of Uruguay'] = 'Uruguay'

country_asr$location[country_asr$location == 'French Republic'] = 'France'

country_asr$location[country_asr$location == "People's Republic of Bangladesh"] = 'Bangladesh'

country_asr$location[country_asr$location == 'Republic of Tajikistan'] = 'Tajikistan'

country_asr$location[country_asr$location == 'Republic of Ecuador'] = 'Ecuador'

country_asr$location[country_asr$location == 'Republic of Korea'] = 'South Korea'

country_asr$location[country_asr$location == 'Commonwealth of Dominica'] = 'Dominica'

country_asr$location[country_asr$location == 'Republic of Poland'] = 'Poland'

country_asr$location[country_asr$location == 'Brunei Darussalam'] = 'Brunei'

country_asr$location[country_asr$location == 'Kingdom of Cambodia'] = 'Cambodia'

country_asr$location[country_asr$location == "Antigua and Barbuda"] = 'Lands Antigua'

a <- country_asr[country_asr$location == "Lands Antigua",]

a$location <- 'Barbuda'

country_asr <- rbind(country_asr,a)

country_asr$location[country_asr$location == 'Republic of Uzbekistan'] = 'Uzbekistan'

country_asr$location[country_asr$location == 'Republic of Chile'] = 'Chile'

country_asr$location[country_asr$location == 'Republic of Austria'] = 'Austria'

country_asr$location[country_asr$location == "Democratic People's Republic of Korea"] = 'North Korea'

country_asr$location[country_asr$location == 'Lebanese Republic'] = 'Lebanon'

country_asr$location[country_asr$location == 'Kingdom of Sweden'] = 'Sweden'

country_asr$location[country_asr$location == "Saint Kitts and Nevis"] = 'Saint Kitts'

a <- country_asr[country_asr$location == "Saint Kitts",]

a$location <- 'Nevis'

country_asr <- rbind(country_asr,a)

country_asr$location[country_asr$location == 'Republic of Colombia'] = 'Colombia'

country_asr$location[country_asr$location == 'Republic of Honduras'] = 'Honduras'

country_asr$location[country_asr$location == "Saint Vincent and the Grenadines"] = 'Saint Vincent'

a <- country_asr[country_asr$location == "Saint Vincent",]

a$location <- 'Grenadines'

country_asr <- rbind(country_asr,a)

country_asr$location[country_asr$location == "People's Republic of China"] = 'China'

country_asr$location[country_asr$location == 'Republic of Armenia'] = 'Armenia'

country_asr$location[country_asr$location == 'Republic of Singapore'] = 'Singapore'

country_asr$location[country_asr$location == 'Republic of Zimbabwe'] = 'Zimbabwe'

country_asr$location[country_asr$location == 'Independent State of Samoa'] = 'Samoa'

country_asr$location[country_asr$location == 'Kingdom of Bahrain'] = 'Bahrain'

country_asr$location[country_asr$location == 'Republic of Albania'] = 'Albania'

country_asr$location[country_asr$location == 'Republic of Belarus'] = 'Belarus'

country_asr$location[country_asr$location == "United Kingdom of Great Britain and Northern Ireland"] = 'UK'

country_asr$location[country_asr$location == 'Republic of Mozambique'] = 'Mozambique'

country_asr$location[country_asr$location == 'Republic of Bulgaria'] = 'Bulgaria'

country_asr$location[country_asr$location == 'Republic of Peru'] = 'Peru'

country_asr$location[country_asr$location == 'Republic of Fiji'] = 'Fiji'

country_asr$location[country_asr$location == 'Republic of Malta'] = 'Malta'

country_asr$location[country_asr$location == 'Gabonese Republic'] = 'Gabon'

country_asr$location[country_asr$location == 'Republic of Guinea'] = 'Guinea'

country_asr$location[country_asr$location == 'Republic of Liberia'] = 'Liberia'

country_asr$location[country_asr$location == 'Republic of Sudan'] = 'Sudan'

country_asr$location[country_asr$location == 'Kingdom of Morocco'] = 'Morocco'

country_asr$location[country_asr$location == 'Federal Republic of Germany'] = 'Germany'

country_asr$location[country_asr$location == 'Republic of Nicaragua'] = 'Nicaragua'

country_asr$location[country_asr$location == 'Trinidad and Tobago'] = 'Trinidad'

a <- country_asr[country_asr$location == "Trinidad",]

a$location <- 'Tobago'

country_asr <- rbind(country_asr,a)

country_asr$location[country_asr$location == 'Kingdom of Denmark'] = 'Denmark'

country_asr$location[country_asr$location == 'Republic of Guyana'] = 'Guyana'

country_asr$location[country_asr$location == 'Kingdom of Eswatini'] = 'Swaziland'

country_asr$location[country_asr$location == 'Republic of Guinea-Bissau'] = 'Guinea-Bissau'

country_asr$location[country_asr$location == 'Republic of Serbia'] = 'Serbia'

country_asr$location[country_asr$location == 'Republic of Iraq'] = 'Iraq'

country_asr$location[country_asr$location == 'Slovak Republic'] = 'Slovak'

country_asr$location[country_asr$location == 'Kingdom of Tonga'] = 'Tonga'

country_asr$location[country_asr$location == 'Republic of Costa Rica'] = 'Costa Rica'

country_asr$location[country_asr$location == 'Republic of Rwanda'] = 'Rwanda'

country_asr$location[country_asr$location == 'Republic of Cyprus'] = 'Cyprus'

country_asr$location[country_asr$location == 'Kingdom of Norway'] = 'Norway'

country_asr$location[country_asr$location == 'Republic of Equatorial Guinea'] = 'Equatorial Guinea'

country_asr$location[country_asr$location == 'Republic of Iceland'] = 'Iceland'

country_asr$location[country_asr$location == 'Sultanate of Oman'] = 'Oman'

country_asr$location[country_asr$location == 'Republic of Guatemala'] = 'Guatemala'

country_asr$location[country_asr$location == 'Kingdom of the Netherlands'] = 'Netherlands'

country_asr$location[country_asr$location == 'Commonwealth of the Bahamas'] = 'Bahamas'

country_asr$location[country_asr$location == 'Republic of the Congo'] = 'Republic of Congo'

country_asr$location[country_asr$location == 'Republic of Maldives'] = 'Maldives'

country_asr$location[country_asr$location == 'Republic of Mauritius'] = 'Mauritius'

country_asr$location[country_asr$location == 'Democratic Republic of Timor-Leste'] = 'Timor-Leste'

country_asr$location[country_asr$location == 'United States of America'] = 'USA'

country_asr$location[country_asr$location == 'Taiwan (Province of China)'] = 'Taiwan'

country_asr$location[country_asr$location == 'Democratic Socialist Republic of Sri Lanka'] = 'Sri Lanka'

country_asr$location[country_asr$location == 'Republic of South Africa'] = 'South Africa'

country_asr$location[country_asr$location == 'Republic of Slovenia'] = 'Slovenia'

country_asr$location[country_asr$location == 'Republic of Malawi'] = 'Malawi'

country_asr$location[country_asr$location == 'Republic of Turkey'] = 'Turkey'

country_asr$location[country_asr$location == 'Republic of Haiti'] = 'Haiti'

country_asr$location[country_asr$location == 'Republic of Croatia'] = 'Croatia'

country_asr$location[country_asr$location == 'Republic of Azerbaijan'] = 'Azerbaijan'

country_asr$location[country_asr$location == 'State of Kuwait'] = 'Kuwait'

country_asr$location[country_asr$location == 'Republic of Yemen'] = 'Yemen'

country_asr$location[country_asr$location == 'Republic of Estonia'] = 'Estonia'

country_asr$location[country_asr$location == 'Kingdom of Thailand'] = 'Thailand'

country_asr$location[country_asr$location == 'United States Virgin Islands'] = 'Virgin Islands'

country_asr$location[country_asr$location == 'Socialist Republic of Viet Nam'] = 'Vietnam'

country_asr$location[country_asr$location == 'Kingdom of Spain'] = 'Spain'

country_asr$location[country_asr$location == 'Republic of Finland'] = 'Finland'

country_asr$location[country_asr$location == 'Portuguese Republic'] = 'Portugal'

country_asr$location[country_asr$location == 'Republic of Madagascar'] = 'Madagascar'

country_asr$location[country_asr$location == 'Republic of India'] = 'India'

country_asr$location[country_asr$location == 'Republic of Seychelles'] = 'Seychelles'

country_asr$location[country_asr$location == 'Republic of Mali'] = 'Mali'

country_asr$location[country_asr$location == 'Republic of Namibia'] = 'Namibia'

country_asr$location[country_asr$location == 'Principality of Monaco'] = 'Monaco'

country_asr$location[country_asr$location == 'Swiss Confederation'] = 'Switzerland'

country_asr$location[country_asr$location == 'Hellenic Republic'] = 'Greece'

country_asr$location[country_asr$location == 'State of Qatar'] = 'Qatar'

country_asr$location[country_asr$location == 'Kingdom of Saudi Arabia'] = 'Saudi Arabia'

country_asr$location[country_asr$location == 'Republic of San Marino'] = 'San Marino'

country_asr$location[country_asr$location == 'Republic of Kiribati'] = 'Kiribati'

country_asr$location[country_asr$location == 'Federal Democratic Republic of Ethiopia'] = 'Ethiopia'

country_asr$location[country_asr$location == 'State of Israel'] = 'Israel'

country_asr$location[country_asr$location == 'Republic of Suriname'] = 'India'

country_asr$location[country_asr$location == 'United Republic of Tanzania'] = 'Tanzania'

country_asr$location[country_asr$location == "Lao People's Democratic Republic"] = 'Laos'

country_asr$location[country_asr$location == 'Republic of Ghana'] = 'Ghana'

country_asr$location[country_asr$location == 'Islamic Republic of Iran'] = 'Iran'

country_asr$location[country_asr$location == 'Republic of Indonesia'] = 'Indonesia'

country_asr$location[country_asr$location == 'United Mexican States'] = 'Mexico'

country_asr$location[country_asr$location == 'Republic of Nauru'] = 'Nauru'

country_asr$location[country_asr$location == 'Republic of Tunisia'] = 'Tunisia'

country_asr$location[country_asr$location == 'Federal Democratic Republic of Nepal'] = 'Nepal'

country_asr$location[country_asr$location == 'Islamic Republic of Pakistan'] = 'Pakistan'

country_asr$location[country_asr$location == 'Republic of Senegal'] = 'Senegal'

country_asr$location[country_asr$location == 'Islamic Republic of Mauritania'] = 'Mauritania'

country_asr$location[country_asr$location == 'Syrian Arab Republic'] = 'Syria'

country_asr$location[country_asr$location == 'Independent State of Papua New Guinea'] = 'Papua New Guinea'

country_asr$location[country_asr$location == 'Republic of the Marshall Islands'] = 'Marshall Islands'

country_asr$location[country_asr$location == 'Union of the Comoros'] = 'Comoros'

country_asr$location[country_asr$location == 'Kyrgyz Republic'] = 'Kyrgyzstan'

country_asr$location[country_asr$location == "People's Democratic Republic of Algeria"] = 'Algeria'

country_asr$location[country_asr$location == 'Federated States of Micronesia'] = 'Micronesia'

country_asr$location[country_asr$location == 'Hashemite Kingdom of Jordan'] = 'Jordan'

country_asr$location[country_asr$location == 'Republic of Lithuania'] = 'Lithuania'

country_asr$location[country_asr$location == 'Republic of Chad'] = 'Chad'

country_asr$location[country_asr$location == 'Republic of Zambia'] = 'Zambia'

country_asr$location[country_asr$location == 'Republic of South Sudan'] = 'South Sudan'

country_asr$location[country_asr$location == 'Republic of Latvia'] = 'Latvia'

country_asr$location[country_asr$location == 'Republic of the Niger'] = 'Niger'

country_asr$location[country_asr$location == 'Argentine Republic'] = 'Argentina'

country_asr$location[country_asr$location == 'Republic of Kazakhstan'] = 'Kazakhstan'

country_asr$location[country_asr$location == 'Republic of Niue'] = 'Niue'

country_asr$location[country_asr$location == 'Republic of El Salvador'] = 'El Salvador'

country_asr$location[country_asr$location == 'Republic of Benin'] = 'Benin'

country_asr$location[country_asr$location == 'Republic of Burundi'] = 'Burundi'

country_asr$location[country_asr$location == 'Federal Republic of Somalia'] = 'Somalia'

country_asr$location[country_asr$location == 'Republic of Cabo Verde'] = 'Cape Verde'

country_asr$location[country_asr$location == 'Republic of Cuba'] = 'Cuba'

country_asr$location[country_asr$location == 'State of Eritrea'] = 'Eritrea'

country_asr$location[country_asr$location == 'Republic of Palau'] = 'Palau'

country_asr$location[country_asr$location == 'Arab Republic of Egypt'] = 'Egypt'

country_asr$location[country_asr$location == 'Republic of Djibouti'] = 'Djibouti'

country_asr$location[country_asr$location == 'Republic of Cameroon'] = 'Cameroon'

country_asr$location[country_asr$location == 'Republic of Uganda'] = 'Uganda'

country_asr$location[country_asr$location == 'Republic of Moldova'] = 'Moldova'

country_asr$location[country_asr$location == 'Democratic Republic of Sao Tome and Principe'] = 'Sao Tome and Principe'

country_asr$location[country_asr$location == 'Republic of Angola'] = 'Angola'

country_asr$location[country_asr$location == 'Republic of Botswana'] = 'Botswana'

country_asr$location[country_asr$location == 'Republic of Panama'] = 'Panama'

country_asr$location[country_asr$location == 'Federal Republic of Nigeria'] = 'Nigeria'

country_asr$location[country_asr$location == 'Republic of Kenya'] = 'Kenya'

country_asr$location[country_asr$location == "Republic of Côte d'Ivoire"] = 'Ivory Coast'

country_asr$location[country_asr$location == 'Kingdom of Lesotho'] = 'Lesotho'

country_asr$location[country_asr$location == 'Republic of the Gambia'] = 'Gambia'

country_asr$location[country_asr$location == 'Republic of Sierra Leone'] = 'Sierra Leone'

country_asr$location[country_asr$location == 'Türkiye'] = 'Turkey'

country_asr$location[country_asr$location == 'Côte d'Ivoire'] = 'Ivory Coast'

country_asr$location[country_asr$location == 'Iran (Islamic Republic of)'] = 'Iran'

country_asr$location[country_asr$location == 'Lands Antigua'] = 'Antigua'

country_asr$location[country_asr$location == 'Cabo Verde'] = 'Cape Verde'

country_asr$location[country_asr$location == 'Congo'] = 'Republic of Congo'

country_asr$location[country_asr$location == 'Czechia'] = 'Czech Republic'

country_asr$location[country_asr$location == 'Eswatini'] = 'Swaziland'

country_asr$location[country_asr$location == 'Micronesia (Federated States of)'] = 'Micronesia'

country_asr$location[country_asr$location == 'United Kingdom'] = 'UK'

country_asr$location[country_asr$location == 'Venezuela (Bolivarian Republic of)'] = 'Venezuela'

country_asr$location[country_asr$location == 'Viet Nam'] = 'Vietnam'

country_asr$location[country_asr$location == 'Côte d\'Ivoire'] = 'Ivory Coast'

total <- full_join(worldData,country_asr,by = c('region'='location')) %>%

filter(val !='NA')

total <- total %>%

mutate(val2 = cut(total$val,

breaks = c(0,20,40,60,80,100,120,140,160), # 区间分割???

labels = c("0~20", "20~40", "40~60", "60~80", "80~100", "100~120", "120~140", "140~160"), # 匹配的标签，修正了遗漏和错误的区???

include.lowest = TRUE, right = TRUE))

Here are some of the codes used to analyze the SDI regional level of urinary stones in each country worldwide from 1990 to 2021:

library(dplyr)

library(ggplot2)

options(scipen = 200)

setwd("F:\\GBD\\20-54")

WCBA<-read.csv("IHME-GBD_2021_DATA-bcc7c785-1.csv")

region_order <- read.csv("order.csv",header = F)

WCBA$location <- factor(WCBA$location,

levels=region_order$V1,

ordered=TRUE)

unique(WCBA$location)

colnames(WCBA)

#--------------SDI数据读取--------------------

#SDI下载后去掉标题行

#https://ghdx.healthdata.org/record/global-burden-disease-study-2021-gbd-2021-socio-demographic-index-sdi-1950%E2%80%932021

SDI <- read.csv("SDI1990-2021.csv")

#将SDI转换成长形数据：也就是一个地区，一个年份为一行

library(reshape)

SDI <- melt(SDI,id='Location')

SDI$variable<-as.numeric(gsub('\\X',replacement = '',SDI$variable))

colnames(SDI)<-c('location','year','SDI')

unique(SDI$location)

SDI$location[SDI$location == 'Eastern sub-Saharan Africa'] = 'Eastern Sub-Saharan Africa'

SDI$location[SDI$location == 'Central sub-Saharan Africa'] = 'Central Sub-Saharan Africa'

SDI$location[SDI$location == 'Western sub-Saharan Africa'] = 'Western Sub-Saharan Africa'

SDI$location[SDI$location == 'Southern sub-Saharan Africa'] = 'Southern Sub-Saharan Africa'

#-------------------------患病率prevalence与SDI关联------------------------

WCBA<-subset(WCBA,

WCBA$age=='20-54 years' &

WCBA$metric=='Rate' &

WCBA$measure=='Incidence')

WCBA <- WCBA[,c(2,7,8)]

WCBA_SDI <- merge(WCBA,SDI,by=c('location','year'))

#皮尔逊相关性系数

cor<-cor.test(WCBA_SDI$val,WCBA_SDI$SDI,method = 'pearson')

cor

**The code used is reproducible**

**Results**

1. **Dense presentation:** Results are presented in a dense format with little interpretation, making it difficult for readers to extract key messages.

**Re：**Thanks to the reviewers for their comments, we have summarized the conclusions in concise language at the end of each result.

2. **Limited focus on practical insights:** While the numbers are well-detailed, there’s insufficient focus on what these trends mean in a practical context.

**Re：**Thanks to the reviewers for their comments, we have summarized the conclusions in concise language at the end of each result, and the possible reasons for this have been suggested

3. **Underutilization of visuals:** Figures and tables are used but could be more impactful if tailored to highlight key trends and findings.

**Re：**Thanks for the reviewer's comments, we have drawn the correlation diagram based on the data results at the end of each result, and the pictures and notes are at the end of the document.

**Discussion**

1. **Lack of critical analysis:** While results are discussed, there’s limited critical engagement with potential biases or alternative explanations for the findings.

**Re：**Thanks to the reviewers for their comments on the manuscript, I have added some critical analysis of the results at the end of the discussion

2. **Overgeneralization of results:** Some conclusions, such as the need for "focused intervention techniques," are too broad without specifying actionable recommendations.

**Re：**Thanks for the reviewer's comments. As for the over-generalization and over-broad results, I have made a supplement in the conclusion of the abstract and the discussion part of the main text, and tried to make detailed suggestions within the achievable scope

3. **Insufficient limitations:** The study acknowledges data limitations but doesn’t address other potential weaknesses, such as reliance on global datasets or differences in healthcare systems.

**Re：**Thanks to the reviewers for their comments on the manuscript, I add some limited analysis of the conclusions of the analysis results at the end of the discussion

Response to Reviewers#2

1. Abstract
   • The ethical consideration is not specified.

**Re**：Thank you for the reviewer's abstract review comments on the article. The data for our research analysis is sourced from the public database GBD (Global Burden of Disease), and patient data is all from the public database. Everyone can freely browse and download it, and there are no ethical issues.

Response to Reviewers#3

1. In figure 2 (below line 214) incidence rate in the table written in a wrong way.

**Re:** Thank you for your review. I have noticed this problem and revised the spelling of incidence rate in table2.

2. The figures clarity should be adjusted.

**Re:** Thank you for your review. I have revised the figure clarity

3. Discussion: the aim and findings of the result was repeated in details in the discussion, instead it will be better to compare the result and justify the increase in prevalence in more details.
1. **Re:** Dear Reviewer, Thank you for your thorough review and valuable suggestions. In response to your feedback, we have revised the Discussion section as follows: **Strengthened Prevalence Increase Validation**
   1) Incorporated environmental factor analysis.
   2) Addressed diagnostic technology evolution.

Response to Reviewers#4

1. Abstract
   • The ethical consideration is not specified.

**Re**：Thank you for the reviewer's abstract review comments on the article. The data for our research analysis is sourced from the public database GBD (Global Burden of Disease), and patient data is all from the public database. Everyone can freely browse and download it, and there are no ethical issues.

Response to Reviewers#5

1. The title should indicate the focus on epidemiological trends (e.g., "Epidemiological Trends in Urolithiasis Among Working-Age Populations...").

**Re**：We sincerely appreciate the reviewer's constructive feedback regarding the article title. In response to your suggestions, we have revised the title to:
**"Epidemiological Trends of Urolithiasis in Working-Age Populations: Findings from the Global Burden of Disease Study 1990–2021"**
This modification emphasizes our focus on analyzing global disease burden patterns and aligns with the scope of the GBD methodology.

2. Why was the age range of 20–54 chosen? Given that urolithiasis also affects individuals outside this range, a justification is necessary. Were trends in younger (<20) and older (>54) individuals considered?

**Re：**We selected the 20–54 age range for focused analysis based on three key considerations: According to literature review, the average working age in the United States is 25-64 years old（**Wiemers EE, Monnat SM, Wolf DA, Montez JK, Grove J, Gutin I, Grossman E. States' COVID-19 policy contexts and suicide rates among US working-age adults. Health Aff Sch. 2025 Mar 15;3(3):qxaf024. doi: 10.1093/haschl/qxaf024. PMID: 40092402; PMCID: PMC11909630.**）, the average working age in Finland is 15-65 years old（**Hökkä M, Aho AL. Negative Life Changes in the Lives of a Working-Age Widows and Widowers and their Families After the Death of a Spouse- a Qualitative Study. Omega (Westport). 2025 Mar 20:302228251328198. doi: 10.1177/00302228251328198. Epub ahead of print. PMID: 40111178.**）, and the average working age in China is 20-54 years old. The growing disease burden among individuals aged 20-54 years presents substantial socioeconomic challenges by directly affecting workforce productivity and increasing healthcare expenditure. Despite a persistent upward trend in this demographic over recent years, existing epidemiological studies have predominantly focused on geriatric populations. This oversight has led to a significant evidence gap concerning comprehensive health assessments for adults aged 20-54, particularly in understanding the long-term effects of chronic conditions on economic productivity.

3. The manuscript mentions joinpoint regression to detect trends. How were these parameters optimized?

**Re：**We thank the reviewers for their comments on the article. The default method of Joinpoint model optimization is Monte Carlo permutation test. Through this test, the optimal number of join points is determined, and the corresponding model is the optimal model. You can refer to the official website of joinpoint [How Joinpoint Selects the Final Model Joinpoint], and the Permutation Test is generally selected. https://surveillance.cancer.gov/help/joinpoint/setting-parameters/method-and-parameters-tab/model-selection-method

4. While the study discusses trends, it implies potential causation (e.g., economic and healthcare improvements in high SDI regions). Were any confounders or alternative explanations considered?

**Re：**We thank the reviewers for their comments on the article. Our study not only examines trends but also takes into account confounding factors and study limitations, such as inherent limitations within the GBD database and variations in data collection quality. Additionally, these potential limitations are addressed in the concluding part of the discussion section.

Response to Reviewers#6

1. Sentence Structure: The clarity of several sentences needs improvement. Additionally, there are instances where the same sentence is repeated. Revising these sections for conciseness and coherence would be beneficial.

**Re：**We thank the reviewers for their comments on the article. In order to use the terminology more precisely. We have changed the "migraine" and "epidemic" in the article to "urolithiasis", which is our negligence in writing. Thank you for your correction.

2. Tables and Figures
   Tables: Certain tables, such as Table 2, are excessively lengthy and may overwhelm the reader. Consider condensing these tables to present the most critical data.
   Figures: The quality of the figures needs enhancement. Many figures appear blurry and require higher resolution to ensure clarity and readability.

**Re：**We thank the reviewers for their comments on the article. We have modified the figure definition according to the requirements of the journal. In this study, we believe that the current presentation format is not conducive to further compression due to the following reasons: This is a comprehensive report on the global burden of disease, which retains the absolute values and change rates of prevalence, incidence, and DALYs from 1990 to 2021. These data are essential for fulfilling the requirements of four-dimensional analysis in transnational health transitions:

(1) cross-validation of multi-dimensional indicators (e.g., analyzing the paradox of declining incidence but increasing prevalence);

(2) attribution of long-term trends (a 31-year span requires baseline value support);

(3) Only through comparisons among all 204 countries can extreme value contrasts be achieved (e.g., Brazil’s +94% versus the United States’ -50%, which must be presented independently).

Therefore, we believe that we should try to avoid compressing table2 to facilitate the comparative analysis of readers.

3. Data Analysis
   Use of Absolute Numbers: While absolute numbers are acceptable, they may not accurately reflect changes over time when comparing data. It is important to consider population size when analyzing trends. For example, 3 cases in a population of 100 are proportionally more significant than 5 cases in a population of 1000.

**Re：**The point you mentioned is very important! Absolute numbers, while intuitive, do obscure the true picture when they lack a frame of reference. The difference of population base and the change of time dimension were the key factors affecting the interpretation of the data. We thank the reviewers for their comments on the article. The absolute numbers we use in this article are all derived from the GBD database, and the absolute numbers can sometimes reflect the absolute changes of the data over the years, which we believe is also valuable. When describing the changing trend, we follow your suggestion to use the proportion of the same situation to reflect the changing trend in the article.
