# Supplementary material for: Epidemiological trends of urolithiasis in working-age populations: Findings from the global burden of disease study 1990–2021
Source: PLoS One. 2025 Jul 1;20(7):e0327343. doi: 10.1371/journal.pone.0327343 (PMC12212516; doi:10.1371/journal.pone.0327343)
Supplement: Attachment — Submitted filename: Rebuttal Letter.docx [file pone.0327343.s017.docx]

**Rebuttal Letter**

**Editor:**

Upon careful examination, it has come to my attention that there are several areas within your manuscript that require attention. Specifically, the formatting, figures, tables, and language used in the document appear to be somewhat disorganized and contain a number of errors. These issues detract from the overall quality and clarity of your research presentation.
To enhance the manuscript's chances of acceptance and to align with the standards of the journal, I strongly recommend that you undertake a thorough revision. This should include:

**Comment：**1. Formatting: Ensuring that the manuscript adheres strictly to the guidelines provided in the journal. This includes the structure, headings, font, margins, and citation style.
2. Figures and Tables: Reviewing all figures and tables for clarity, relevance, and accuracy. Each should be properly labeled, captioned, and referenced within the text.
3. Language and Grammar: Engaging a professional language editor or a native English speaker to refine the language and correct grammatical errors. This will significantly improve the readability and professionalism of your manuscript.

**Response :** 1.Thank you for your advice and I have ensured that the manuscript strictly follows the guideline format provided by the journal.

2.And check all charts and tables for clarity, relevance, and accuracy. Each is given an appropriate label, title, and reference in the text.

3.I improved the language and corrected the grammatical errors. So that reviewers can better review the manuscript.
